# Identification of Metabolically Quiescent *Leishmania mexicana* Parasites in Peripheral and Cured Dermal Granulomas Using Stable Isotope Tracing Imaging Mass Spectrometry

**DOI:** 10.1128/mBio.00129-21

**Published:** 2021-04-06

**Authors:** Joachim Kloehn, Berin A. Boughton, Eleanor C. Saunders, Sean O’Callaghan, Katrina J. Binger, Malcolm J. McConville

**Affiliations:** a Department of Biochemistry and Molecular Biology, Bio21 Institute of Molecular Science, University of Melbourne, Parkville, Victoria, Australia; b Metabolomics Australia, University of Melbourne, Parkville, Victoria, Australia; c Department of Biochemistry and Genetics, La Trobe Institute for Molecular Sciences, La Trobe University, Bundoora, Victoria, Australia; Harvard T. H. Chan School of Public Health

**Keywords:** drug resistance mechanisms, granuloma, heavy water labelling, intracellular pathogens, leishmaniasis, microbial metabolism, persistence, protists

## Abstract

*Leishmania* are sandfly-transmitted protists that induce granulomatous lesions in their mammalian host. Although infected host cells in these tissues can exist in different activation states, the extent to which intracellular parasites stages also exist in different growth or physiological states remains poorly defined. Here, we have mapped the spatial distribution of metabolically quiescent and active subpopulations of Leishmania mexicana in dermal granulomas in susceptible BALB/c mice, using *in vivo* heavy water labeling and ultra high-resolution imaging mass spectrometry. Quantitation of the rate of turnover of parasite and host-specific lipids at high spatial resolution, suggested that the granuloma core comprised mixed populations of metabolically active and quiescent parasites. Unexpectedly, a significant population of metabolically quiescent parasites was also identified in the surrounding collagen-rich, dermal mesothelium. Mesothelium-like tissues harboring quiescent parasites progressively replaced macrophage-rich granuloma tissues following treatment with the first-line drug, miltefosine. In contrast to the granulomatous tissue, neither the mesothelium nor newly deposited tissue sequestered miltefosine. These studies suggest that the presence of quiescent parasites in acute granulomatous tissues, together with the lack of miltefosine accumulation in cured lesion tissue, may contribute to drug failure and nonsterile cure.

## Abstract (precis)

Many microbial pathogens switch between different growth and physiological states *in vivo* in order to adapt to local nutrient levels and host microbicidal responses. Heterogeneity in microbial growth and metabolism may also contribute to nongenetic mechanisms of drug resistance and drug failure.

## INTRODUCTION

Microbial pathogens can adapt to local changes in the tissue microenvironment, such as nutrient gradients, hypoxia, and host microbicidal effectors, by switching between different growth and metabolic states (1). The ability of some pathogens to switch between a range of different growth and metabolic phenotypes *in vivo* may allow them to resist or avoid host microbicidal responses and establish long term chronic infections. Heterogeneity in the metabolic or growth status of microbial pathogens *in vivo* may also confer resistance to many antibiotics and antimicrobial drugs, leading to nonsterile cure and the subsequent acquisition of genetically encoded drug resistance (2–5). However, the measurement of microbial heterogeneity *in vivo* remains challenging. In this study, we have used a universal stable isotope labeling approach coupled with ultra high-resolution imaging mass spectrometry (IMS) to detect metabolically distinct subpopulations of Leishmania mexicana protists in acutely infected animal tissue and define the physiological state of parasite populations that persist after drug treatment.

*Leishmania* spp. are sandfly-transmitted parasitic protists that cause a spectrum of diseases in humans ranging from localized cutaneous lesions to disfiguring mucocutaneous and lethal visceral infections (6). More than 12 million people have acute infections, with 700,000 to 1 million new cases each year (7, 8). An additional 120 million people may be chronically infected with *Leishmania*, facilitating transmission, as well as presenting a risk for disease relapse in immunocompromised individuals (9). Current drug treatments for human leishmaniasis are limited and commonly fail to achieve sterile cure, with surviving parasites contributing to disease reactivation (6, 10). Particularly high rates of relapse have been observed with miltefosine (hexadecylphosphocholine), the only orally administered drug approved to treat leishmaniasis (11, 12). Miltefosine treatment failure has so far not been linked to genetic changes, indicating that a subpopulation of parasites may acquire phenotypic resistance *in vivo* and/or that miltefosine may not reach lethal concentrations in certain tissue niches (13–15). Understanding the causes and consequences of this treatment failure is crucial to ultimately developing drug regimens which achieve a sterile cure.

A hallmark of all *Leishmania* infections is the formation of inflammatory lesions or granulomas, comprising large aggregates of infected and uninfected macrophages and other immune cells (10, 16–18). Granulomas generally arise at or near the site of the sandfly bite or can occur in distal tissues, including the liver and spleen in the case of viscerotropic species (16, 19). The formation of *Leishmania* dermal granulomas is initiated by parasite infection of neutrophils and tissue-resident macrophages in the skin, followed by the recruitment of inflammatory monocytes, as well as neutrophils, eosinophils, and lymphocytes (19–21). *Leishmania* spp. primarily infect macrophages and monocytes in developing granulomas, proliferating as amastigote stages within a vacuolar compartment that contains all the markers of a mature phagolysosome (22). Although *Leishmania* granulomas generally lack a caseous core and have a more homogeneous structure than granulomas induced by other pathogens, such as *Mycobacteria tuberculosis* (23), substantial microheterogeneity is likely to occur within these tissues due to variability in the origin and activation state of macrophages and monocytes (18). For example, the development of a dominant CD4^+^-dependent T-helper 1 cells (TH1) host immune response, with local production of interleukin-12 (IL-12), gamma interferon, and tumor necrosis factor alpha, can lead to polarization of granuloma monocytes/macrophages toward a proinflammatory M1 phenotype, with increased expression of iNOS and production of nitrous oxide (NO) and concomitant restriction of parasite growth (20, 24). Conversely, development of a T-helper 2 cell (TH2) response with production of anti-inflammatory cytokines (IL-4, IL-13, and IL-10) leads to the polarization of granuloma macrophages toward a more permissive M2 phenotype and continued expansion of parasite burden in lesions. Strikingly, recent studies have shown that both M1 and M2 polarized macrophages can coexist in the same tissues, even in the presence of a strong TH1 response due to local production of IL-4/IL-13 by granuloma eosinophils (21). Other factors, such as gradients of diffusible NO (25, 26), hypoxia (27), or salt (28) and nutrient levels, can also modulate host cell polarization, although the extent to which variability in host cell activation leads to heterogeneity in parasite growth and metabolism *in vivo* remains poorly defined.

A number of approaches have been used to assess *Leishmania* heterogeneity in granulomatous tissues, including the use of genetically encoded fluorescent protein reporters (25, 29), or *in vivo* labeling with 5-bromo-2′-deoxyuridine (BrdU) (30). However, these approaches report on single cellular parameters (synthesis of a single protein or DNA replication) and require the generation of transgenic parasite strains or labeling of infected animals with compounds such as BrdU. We have recently developed a complementary universal labeling approach for measuring key cellular parameters in *Leishmania* using heavy water (^2^H_2_O) labeling (31). In the presence of low concentration of ^2^H_2_O, deuterium (^2^H) is incorporated into a wide range of metabolites, including nucleotides, amino acids, sugars, and fatty acids, that, in turn, are precursors for membrane lipids and macromolecules. ^2^H_2_O rapidly equilibrates across tissues and membranes and can be used to measure rates of DNA, RNA, and protein turnover, as well as key metabolic pathways (carbohydrate and lipid synthesis) in intracellular parasite stages in acute-phase granulomas (31–33). Using this approach, we have shown that the bulk population of L. mexicana amastigotes in granulomatous tissues have a very low growth rate (half-life [*t*_1/2_], ∼12 days) and switch to a metabolically quiescent state, characterized by low rates of RNA, protein, and lipid synthesis (31). However, these studies did not provide information on the extent to which parasite metabolism and growth vary spatially within these tissues. In this study, we have combined ^2^H_2_O-labeling with matrix-assisted laser desorption ionization/Fourier transform ion cyclotron resonance/imaging mass spectrometry (MALDI-FTICR-IMS) to identify and map the distribution of distinct subpopulations of L. mexicana amastigotes in murine granulomatous tissues. MALDI-FTICR-IMS allowed high spatial resolution quantitation of levels of ^2^H enrichment in parasite and host-derived lipids in murine granulomas, which was used to identify a patchwork of metabolically active and metabolically quiescent parasite populations within these tissues. We also identified, for the first time, a distinct population of quiescent parasites in the collagen-rich mesothelium basement membrane that underlies dermal granulomas. Significantly, MALDI-FTICR-IMS revealed that miltefosine accumulates in macrophage-rich granuloma tissues but not in the collagen-rich tissues that expand during lesion healing. These latter tissues contained metabolically quiescent parasites, suggesting that resolution of granulomas may lead to the formation of privileged sites which do not accumulate drug and maintain parasites in a quiescent state. This approach can be readily adapted to study spatial and temporal heterogeneity in the metabolism of other microbial pathogens and their responses to drugs *in vivo*.

## RESULTS

### Identification of parasite and host-specific lipids in BALB/c lesions.

**FIG 1:**
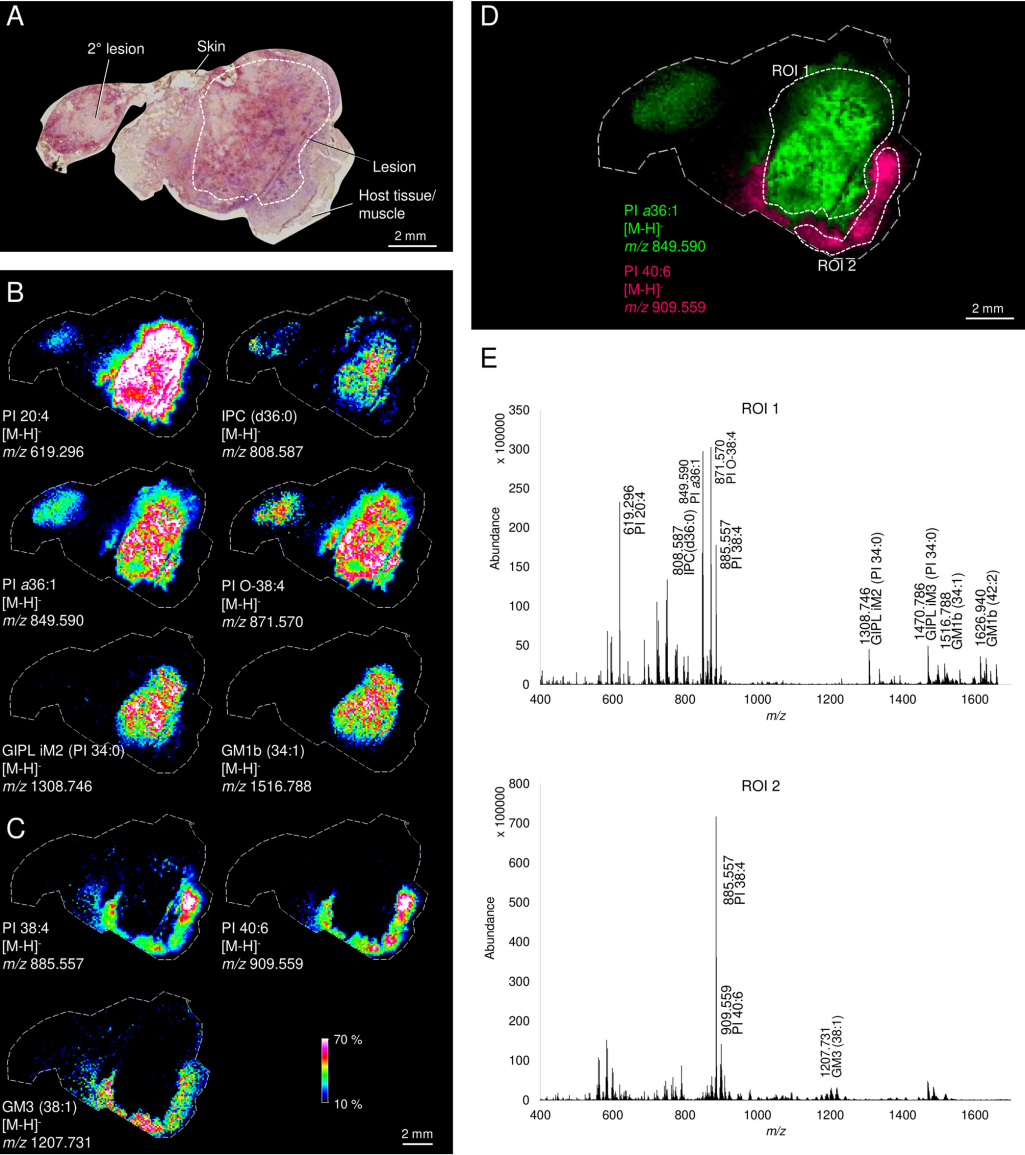
Spatial distribution of *Leishmania* and host-specific phospho-/glycolipids in acute dermal granulomas. Granulomatous lesions were isolated from L. mexicana-infected BALB/c mice (7 weeks postinfection), and consecutive frozen sections were processed for H&E histology or imaging by MALDI-FTICR-MS in negative-ion mode. (A) H&E-stained longitudinal section through lesion with overlying skin and surrounding muscle tissue. (B) Spatial distribution of parasite- and host-specific lipids. The parasite-specific lipids are IPC d36:0 (*m/z* 808.587), PI O-38:4 (*m/z* 871.570), PI a36:1 (*m/z* 808.587), and GIPL species, Man_2_GlcN-PI (iM2, *m/z* 1,308.746). The host-specific lipids are GM1b 34:1 (*m/z* 1,516.788) and lyso-PI 20:4 (*m/z* 619.296). (C) Spatial distribution of host lipid species that localized to surrounding tissue (PI 38:4 (*m/z* 885.557); PI 40:6 (*m/z* 909.559), and GM3 38:1 (*m/z* 1,207.731). (D) Overlay of lipid markers unique to lesion (PI *a*36:1) and nonlesion (PI 40:6) tissues. (E) Average mass spectra of pixels in designated ROIs (ROI 1, lesion; ROI 2, surrounding muscle tissue) in panel D as determined by MALDI-FTICR-MS. Heat map distributions of lipids are displayed at 10 to 70% intensity (percentage of the maximum).

Infection of BALB/c mice with L. mexicana promastigotes leads to the development of large, unstructured granuloma-like inflammatory lesions at the site of infection. In contrast to granulomas induced by some other pathogens (23), L. mexicana granulomas lack a conspicuous margin or an acellular caseous center (see Fig. S1A and B in the supplemental material). Flow cytometry analysis of collagenase-treated granulomas confirmed that these lesions primarily contained infected and uninfected monocytes/macrophages, neutrophils, and T cells (see Fig. S1C) (21, 22). In order to determine whether we could detect parasite and host signature lipids in these acute-phase lesions, excised granulomas were rapidly frozen and consecutive sections through the center of the lesion were stained with either hematoxylin and eosin (H&E) (Fig. 1A) or imaged by MALDI-FTICR-IMS (Fig. 1B to D). Metabolites in the mass range *m/z* 400 to 2,000 were detected in negative ion mode with a pixel spacing of 100 µm. High-density mass spectra (>10,000 mass features) were collected, allowing the detection of metabolites, primarily lipid species that are either unique to L. mexicana amastigotes or to host cells in the granuloma. The major parasite-specific lipids identified in negative ion mode, included well-characterized *Leishmania*-specific lipids such as inositol phosphorylceramide (IPC d36:0) (34, 35) and the glycoinositol phospholipids (GIPL), Hex_2_HexN_1_alkylacyl-PI (iM2), Hex_3_HexN_1_alkylacyl-PI (iM3), and Hex_4_HexN_1_alkylacyl-PI (iM4) (36). The GIPLs are predominantly located in the parasite plasma membrane and form a tightly packed glycocalyx that protects amastigotes from hydrolytic enzymes in the macrophage phagolysosome compartment (37). The distribution of IPC and GIPLs coincided with the infected host cells (typically containing 10 to 100 parasites), as determined by H&E staining of consecutive sections (Fig. 1A and B). Other lipids that were uniquely associated with granulomas included monacyl-, diacyl-, and alkylacyl-phosphatidylinositol species (lyso-PI 20:4, PI *a*36:1, and PI O-38:4), as well as a number of gangliosides (i.e., GM1b 34:1/42:2). Gangliosides are exclusively synthesized by host cells, although they can become incorporated into the plasma membrane of intracellular amastigotes (37). Other PI molecular species and gangliosides detected in negative-ion mode were either distributed across both infected and uninfected tissues, while enriched in the latter (PI 38:4), or exhibited clear enrichment in tissues surrounding the lesion (PI 40:6, ganglioside GM3 38:1) (Fig. 1C and D). Marked differences in the lipid composition of granuloma (region of interest [ROI] 1) and surrounding tissue (ROI 2) were further supported by averaged mass spectra of selected tissue regions of individual sections (Fig. 1E).

10.1128/mBio.00129-21.1 FIG S1 Tissue organization and immune composition of L. mexicana induced granulomas before and after miltefosine treatment. Tissue sections from uninfected (A) and infected BALB/c mice (B) were stained with H&E or Masson trichrome (to stain collagen). Acute-phase *Leishmania* granulomas span the different dermal layers, including the mesothelium basement membrane that separates the skin from underlying tissues. After 21 days of miltefosine treatment, most of the parasites in the original granuloma mass have been cleared, whereas host cells in the mesothelium remain heavily parasitized. (C) Detail of different sections of granulomas from miltefosine-treated mice highlighting marked differences in cell density and size of parasite vacuoles. (D) Representative flow cytometry analysis of a cutaneous L. mexicana infected lesion compared to a distal cutaneous nonlesion. Infected lesion showed a large increase in leucocyte infiltration (CD45^+^) compared to a nonlesion. This was largely comprised of myeloid cells expressing high levels of CD11b and MHC II, with low-intermediate expression of F4/80 and CD11c. Some T cells (CD3^+^) and Neutrophils (Ly6G^+^ CD11b^hi^) are also evident but to a much lesser extent. Download FIG S1, TIF file, 2.7 MB. Copyright © 2021 Kloehn et al. 2021 Kloehn et al. https://creativecommons.org/licenses/by/4.0/ This content is distributed under the terms of the Creative Commons Attribution 4.0 International license.

To further confirm which lesion-specific lipids were derived from intracellular amastigotes or host cells, infected and uninfected monolayers of BALB/c bone marrow-derived macrophages (BMDMs) were analyzed by MALDI-FTICR-IMS (see Fig. S2). Parasite-specific lipids, including PI *a*36:1, IPC d36:0 and GIPL species (iM2) were only detected in parasite-infected BMDM monolayers (see Fig. S2B, C, and E), consistent with parasite origin, while several abundant PI species (PI 38:4, PI 40:5) and gangliosides (GM1b d34:1/d42:2) were detected in both infected and uninfected BMDM monolayers (see Fig. S2A, D, and F). The latter were also detected in uninfected J774 macrophage cell pellets, but not in L. mexicana promastigotes, indicating that they may be general markers of murine macrophages (see Fig. S3A and B) (38). In contrast, IPC and GIPLs were dominant glycolipid species in L. mexicana promastigotes but absent in J774 macrophages (see Fig. S3A and B). As expected, several lipid species, such as PI 36:0, that are intermediates in common lipid biosynthetic pathways were found in both L. mexicana promastigotes and in uninfected macrophages.

10.1128/mBio.00129-21.2 FIG S2 Analysis of lipids of L. mexicana-infected and -uninfected BMDMs. Monolayers of noninfected and infected BMDMs were washed, freeze dried, and coated with matrix prior to analysis by MALDI-FTICR-IMS in negative mode at 100-μm pixel spacing. (A) Total mass spectra of uninfected and infected BMDM monolayers. Major lipids detected include PI 38:4 (*m/z* 885.557) and the gangliosides GM1b (d42:2, *m/z* 1,626.940). All detected ions correspond to the pseudomolecular [M-H]^−^ ion of the indicated lipids. (B to F) Selected [M-H]^−^ ions and corresponding images. Lipid species that were uniquely detected in infected (right panel) but not in noninfected BMDMs (left panel) included IPC d36:0 (*m/z* 808.582; B), PI *a*36:1, (*m/z* 849.590; C), and iM2 GIPL (*m/z* 1,232.569; E). Lipids that were detected in both infected and uninfected BMDMs were PI 38:4 (*m/z* 885.555; D) and GM1b d42:2 (*m/z* 1,626.940; F). All lipids are displayed at 0 to 30% intensity (percentage of the maximum). Download FIG S2, TIF file, 1.8 MB. Copyright © 2021 Kloehn et al. 2021 Kloehn et al. https://creativecommons.org/licenses/by/4.0/ This content is distributed under the terms of the Creative Commons Attribution 4.0 International license.

10.1128/mBio.00129-21.3 FIG S3 Analysis of L. mexicana promastigote and J774 macrophage lipids by MALDI-FTICR-MS. Cell pellets of L. mexicana promastigotes (log growth phase) and J774 macrophages were rapidly frozen and thin sections analyzed by MALDI-FTICR-IMS in negative-ion mode following matrix application. (A) Mass spectra of L. mexicana promastigote lipids (upper panel), with detail of the mass spectra in the lower two panels. (B) Mass spectrum of J774 lipids (upper panel), with detail in lower two panels. The major negatively charged lipids in *Leishmania* promastigotes are PIs, IPCs, and the free glycoinositolphospholipids iM2 (Man_2_GlcN_1_-alkylacyl-PI), iM3 (Man_3_GlcN_1_-alkylacyl-PI), and iM4 (Man_4_GlcN_1_-alkylacyl-PI). All masses correspond to the [M-H]^−^ ions. The PI molecular species PI *a*36:1 (*m/z* 849.590) is abundant in both promastigotes and lesion amastigotes (see Fig. 1). J774 macrophages share several lipid species with BMDM macrophages such as PI 38.4, GM1b (d34:1), and GM1b (d40:1). Other lipid species were predominantly found in J774 macrophages (GM1b d42:1) but not in BMDMs or vice versa (PI 40:5 and GM1b d42:2). Download FIG S3, TIF file, 1.1 MB. Copyright © 2021 Kloehn et al. 2021 Kloehn et al. https://creativecommons.org/licenses/by/4.0/ This content is distributed under the terms of the Creative Commons Attribution 4.0 International license.

Significant differences were also observed in the molecular species composition of parasite lipids extracted from axenically cultivated promastigotes and amastigotes, compared to those extracted from amastigotes that had been isolated from BMDMs or BALB/c lesions. Specifically, the dominant IPC molecular species in axenic parasites were IPC d34:1 and IPC d36:1, while intracellular amastigotes contained predominantly IPC d36:0 (Fig. 1B and E; see also Fig. S2B and S3A), likely reflecting salvage of host ceramide or sphingolipids. Similarly, the major GIPL species in axenic amastigotes/promastigotes contained C_14:0_ or C_12:0_ sn-2 acyl linked fatty acids, while lesion amastigotes contained longer (C_18:0_) sn-2 acyl chains (see Fig. S4). These differences likely reflect the shutdown in sn-2 fatty acid remodeling reactions in lesion amastigotes, which result in the replacement of sn-2 C_18:0_ with C_14:0_ and C_12:0_ sn-2 acyl fatty acids (36). Interestingly, amastigotes isolated from BMDMs appear to retain the fatty acid remodeling as the major GIPL species contained C_12:0_/C_14:0_ acyl chains (see Fig. S2E). Together, these analyzes show that parasite-specific lipids, as well as host-cell specific lipids, are expressed throughout the lesion, readily detected by IMS, and differ markedly from host lipids in surrounding uninfected tissues.

10.1128/mBio.00129-21.4 FIG S4 The major GIPL species of *in vitro* promastigotes and isolated lesion amastigotes have different lipid moieties. Cell pellets of L. mexicana promastigotes (log growth phase) and lesion derived amastigotes were pelleted and imaged by MALDI-FTICR-IMS. The major promastigote GIPL species contain a sn-1 C_18:0_ alkyl chain and either C_14:0_ or C_12:0_ sn-2 acyl chains. In contrast, the amastigote GIPLs contain predominantly sn-1 C_18:0_ alkyl and sn-2 C_18:0_ acyl chains, resulting in a shift of *m/z* 28 in the mass of major molecular species. Lesion amastigotes also contained a number of other GIPL species which were not further characterized (e.g., *m/z* 1,232.575, 1,377.560, 1,394.633, 1,615.799, and 1,643.815). Download FIG S4, TIF file, 0.7 MB. Copyright © 2021 Kloehn et al. 2021 Kloehn et al. https://creativecommons.org/licenses/by/4.0/ This content is distributed under the terms of the Creative Commons Attribution 4.0 International license.

### Measuring host and parasite lipid turnover using ^2^H_2_O labeling and MALDI-FTICR-IMS.

**FIG 2:**
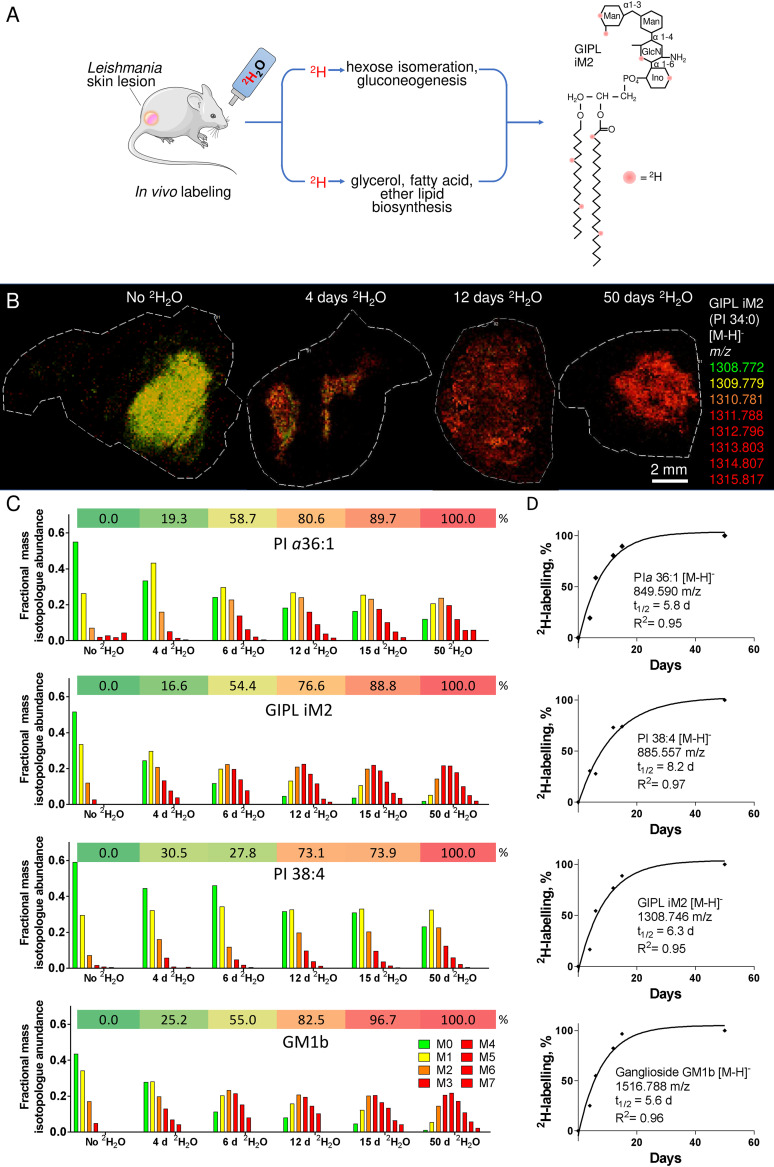
*In vivo* ^2^H labeling of parasite lipids in in infected mice. (A) L. mexicana-infected mice (6 to 9 weeks postinfection) were labeled at 5% ^2^H_2_O in their body water for 4 to 50 days. ^2^H from ^2^H_2_O is incorporated into sugars, glycerol, and lipids during gluconeogenesis, hexose isomerization, and *de novo* glycerol, fatty acid, and ether-lipid biosynthesis, resulting in extensive labeling of phospholipid and glycolipids, such as the GIPL, iM2. (B) Granulomas were excised and analyzed by MALDI-FTICR-MS in negative mode at 100-μm pixel spacing. The different mass isotopologues (M_0_ to M_7_) of the parasite-specific GPI glycolipids, iM2 (*m/z* 1,308.772) are color coded and merged to generate an isotopologue heat map (low mass isotopologues being green/yellow; high mass isotopologues being red). (C) Mass isotopologue (M_0_ to M_8_) distribution of two parasite-specific lipids (PI *a*36:1 and iM2) and two host-specific lipids (PI 38:4 and GM1 34:1) across the lesion section are shown, demonstrating a shift toward higher mass isotopologues with extending duration of ^2^H_2_O labeling. (D) Quantitation of fractional ^2^H enrichment of individual lipids with time and inferred rate of turnover (*t*_1/2_) for each lipid indicated. *R*^2^ values define the goodness of fit.

IPC and the GIPLs are abundant plasma membrane components of all *Leishmania* developmental stages, and the rate of turnover of these lipids likely reflects the rate of cell replication, as well as basal turnover and maintenance of cellular membranes. We have previously shown that ^2^H_2_O labeling can be used to measure the rate of turnover of L. mexicana lipids and glycans in infected BALB/c lesions (31). L. mexicana-infected BALB/c mice were metabolically labeled with ^2^H_2_O over 1 to 50 days and the increase in ^2^H enrichment in parasite and host lipids *in situ* determined by MALDI-FTICR-IMS (Fig. 2A). ^2^H enrichment in parasite IPCs, PIs, and GIPLs was associated with the progressive increase in abundance of an envelope of high mass isotopologues (M1 to M6) (Fig. 2B and C), which reached steady-state between 20 and 50 days (Fig. 2B to D). ^2^H enrichment in macrophage PIs and gangliosides was also observed. The empirically determined ^2^H maximum enrichment value can be used to calculate the half-life (*t*_1/2_) for each lipid. These analyses suggested that the *t*_1/2_ for parasite-specific lipids, such as PI*a*36:1 and iM2 were 5.8 and 6.2 days, respectively (Fig. 2D). This is faster than the previously determined rate of parasite DNA turnover in lesions (*t*_1/2_ of 11.8 days) (31), indicating that bulk plasma membrane lipids are turned over at approximately twice the rate of replication. Interestingly, host-specific lipids, such as GM1b and PI 38:4, also exhibited relatively low rates of turnover (5.6 and 8.2 days, respectively) (Fig. 2D), suggesting that most granuloma host cells also have low rates of proliferation.

To further assess whether ^2^H labeling of amastigote glycolipids primarily reflects labeling of the glycan head group, the lipid moiety or both, labeled isotopomers detected in tissue slices were subjected to tandem mass spectrometry (MS/MS) analysis. ^2^H enrichment was observed in diagnostic fragment ions containing both the glycan and the lipid moieties, consistent with known dependency of amastigotes on both active hexose uptake and catabolism, as well as *de novo* fatty acid biosynthesis for growth *in vivo* (see Fig. S5) (31, 32).

10.1128/mBio.00129-21.5 FIG S5 MS/MS analysis reveals ^2^H enrichment occurs in the lipid tail, as well as the polar head of glycolipids during synthesis. L. mexicana log-phase promastigotes were cultivated in media containing zero (left panel) or 5% ^2^H_2_O (vol/vol) for 24 h (right panel), and cell pellets were imaged by MALDI-FTICR-MS/MS in negative mode. (A and B) Mass spectra (A) and detail of [M-H]^−^ ions of GIPL iM3 (32:0) (B). The indicated precursor masses of unlabeled (*m/z* 1,442.779) and labeled (*m/z* 1,445.850) iM3 (yellow arrow head) were subjected MS/MS fragmentation (C and D). The MS/MS fragmentation peaks indicated ^2^H enrichment in both the polar headgroup (C) and the apolar lipid tail (D). Download FIG S5, TIF file, 1.5 MB. Copyright © 2021 Kloehn et al. 2021 Kloehn et al. https://creativecommons.org/licenses/by/4.0/ This content is distributed under the terms of the Creative Commons Attribution 4.0 International license.

**FIG 3:**
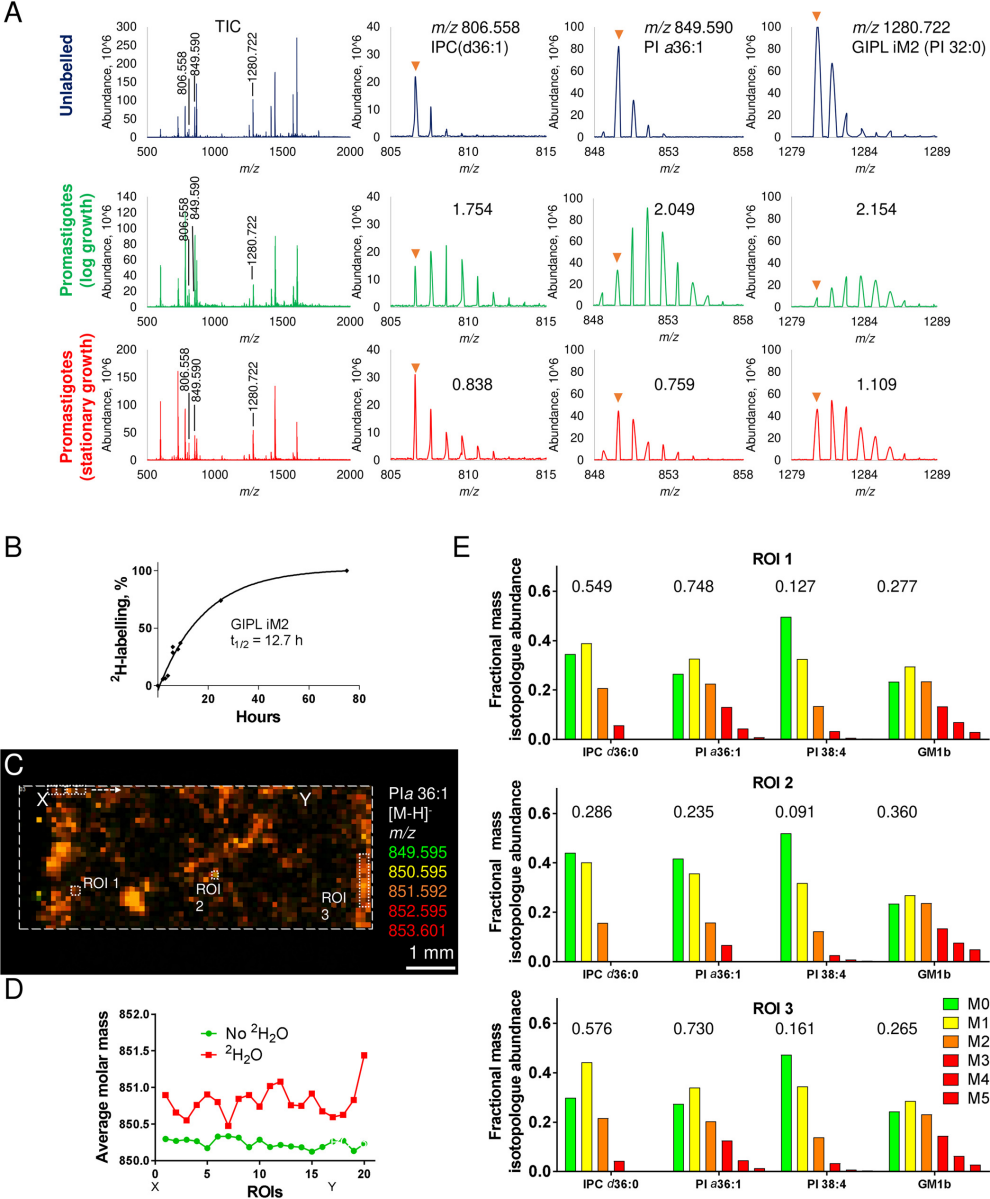
Low-level constitutive lipid turnover in nondividing stages of *Leishmania* and stochastic variability in intracellular amastigotes. (A) L. mexicana promastigotes in log phase or stationary growth phase were cultivated in the presence of 5% ^2^H_2_O for 24 h. Cell pellets were rapidly frozen, sectioned, and scanned by MALDI-FTICR-MS in negative mode. Each panel shows the full mass spectra and select regions of the mass spectrum covering mass isotopologues of IPC (*m/z* 806.558), PI *a*36:1 (*m/z* 849.590), and GIPL iM2 (*m/z* 1,280.722). Mass spectra of cell pellets from unlabeled promastigotes, log-phase promastigotes, and stationary-phase promastigotes are shown. ^2^H enrichments were quantified as the increase in the average mass over natural abundance and are indicated for each stage. (B) ^2^H labeling of GIPL iM2 was measured in log-phase promastigotes over a time course to determine the half-life (*t*_1/2_). (C) BMDMs were infected with L. mexicana promastigotes for 48 h and then labeled with 5% ^2^H_2_O for a further 96 h before being imaged by MALDI-FTICR-MS. The spatial distribution of the parasite-specific lipid PI *a*36:1 and corresponding mass isotopologues are merged to generate an isotopologue heat map. (D) Changes in fractional ^2^H enrichment in PI *a*36:1 across 20 consecutive ROIs (indicated by points X to Y on figure). Each ROI was composed of four binned pixels (100 × 100-μm laser spot/200 × 200 data point). (E) Relative abundance of mass isotopologues of two parasite (IPC 36:1 and PI *a*36:1) and two macrophage (PI 38:4 and GM1b) lipids in the three ROI shown in panel C. The levels of ^2^H enrichment are indicated above each mass isotopologue plot.

Finally, to investigate whether the level of ^2^H enrichment in parasite-specific lipids is primarily regulated by growth rate or metabolic status, we compared the rate of turnover of IPC, PI α36:1, and iM2s in dividing (log phase) or nondividing (stationary phase) L. mexicana promastigotes. Both stages are metabolically active (based on glucose uptake and global metabolic fluxes) despite having markedly different growth rates. The rate of labeling of all lipids was markedly reduced in non-dividing promastigotes (Fig. 3A). In the case of iM2, this corresponded to a 5-fold decrease in turnover (12.7 h versus 2.6 days) (Fig. 3B), indicating that ^2^H enrichment in these lipids primarily reflects the rate of promastigote replication. Interestingly, the rate of turnover of iM2 in nondividing promastigotes is still 3-fold faster than in lesion amastigotes (Fig. 2C), which grow slowly but are much less metabolically active than promastigotes. These data suggest that the rate of ^2^H labeling of parasite GIPLs primarily reflects the rate of replication in promastigotes stages but is also strongly influenced by metabolic state in intracellular amastigote stages.

### Evidence for stochastic variability of *L. mexicana* amastigote growth within lesions.

**FIG 4:**
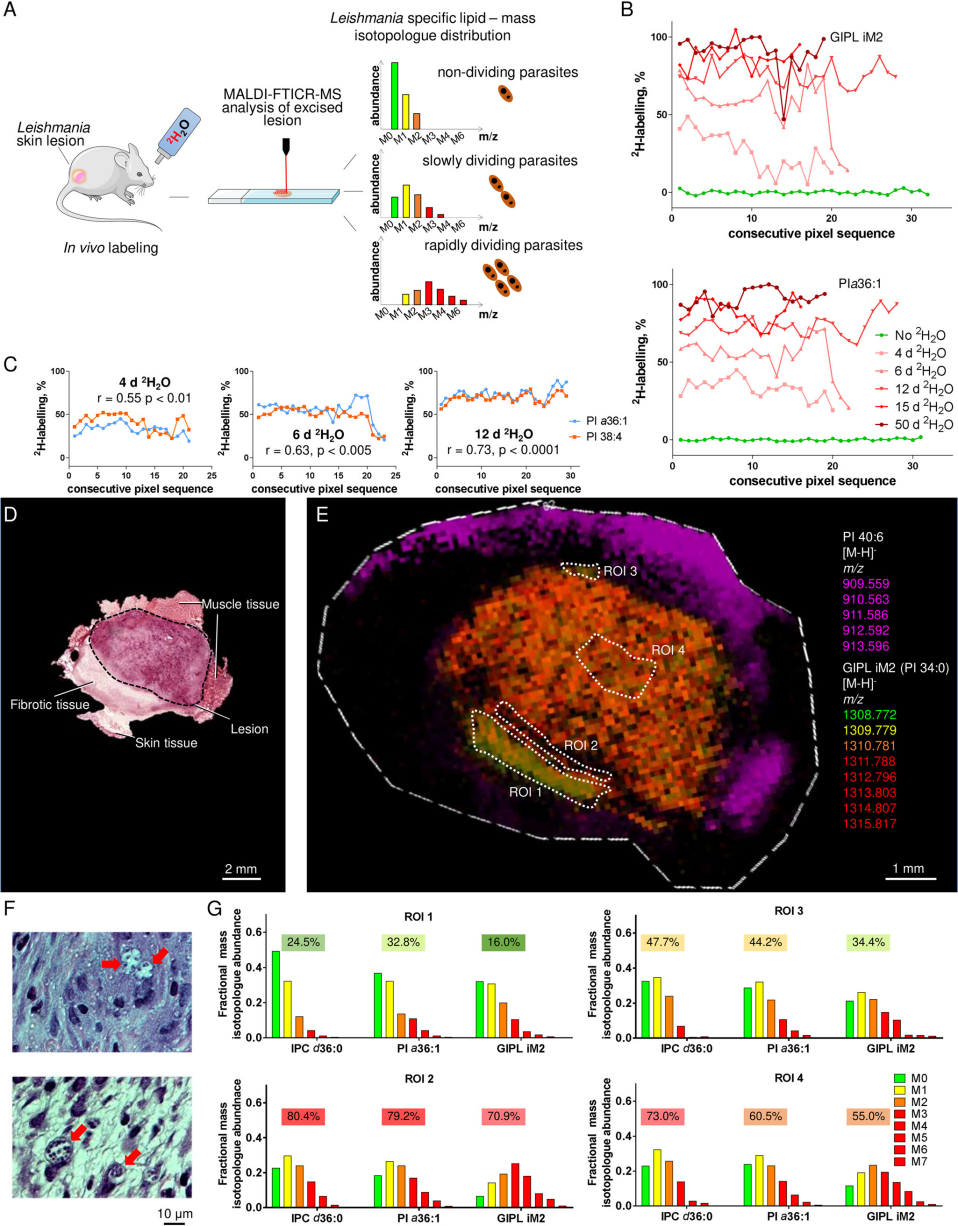
Identification of quiescent populations of L. mexicana amastigotes in regions of lesion fibrosis. (A) Expected isotopomer distributions for parasite lipids labeled *in vivo* in quiescent and actively growing parasite populations. (B) L. mexicana lesions were excised from infected BALB/c mice provided with regular H_2_O (green line) or ^2^H_2_O for 4 to 50 days, and the fractional ^2^H enrichment in two parasite-specific lipids (PI *a*36:1 and GIPL iM2) across transverse sections of different lesions was determined by MALDI-FTICR-MS. Data were extracted from four binned pixels composed of four laser spots (100 × 100-μm laser spot/200 × 200-µm data point). The number of data points varies from 18 to 32, depending on the size of the lesion. (C) Correlation in ^2^H enrichment in the parasite-specific (PI *a*36:1, blue line) and host-specific (PI 38:4; orange line) lipids in transverse sections of granulomas labeled for 4, 6, and 12 days. The correlation coefficient was determined according to the Spearman rank correlation coefficient (no correlation, −1; perfect correlation, +1). (D to G) L. mexicana-infected BALB/c mice were labeled at 5% ^2^H_2_O body water for 6 days, and consecutive sections of granulomatous tissue containing underlying dermal mesothelium were stained with H&E (D and F) or imaged by MALDI-FTICR-MS (E and G). (D) H&E-stained section of granuloma core and surrounding regions of fibrosis (fib, corresponding to mesothelium) and muscle. (E) Spatial distribution of the muscle tissue-specific lipid PI 40:6 (magenta) and the parasite-specific glycolipid iM2 (*m/z* 1,308.772). The different mass isotopologues (M_0_ to M_7_) of iM2 are color coded and merged to generate an isotopologue heat map. (F) Detail of a collagen-rich mesothelium layer containing infected host cells (red arrows). Images were collected from a different lesion that had been chemically fixed for improved imaging. (G) Relative abundances of different mass isotopologues of parasite-specific lipids—IPC d36:0, PI *a*36:1, and GIPL iM2—in the four regions of interest (ROI 1 to ROI 4) indicated in panel E. ROI 1 corresponds to the mesothelium.

We next assessed whether MALDI-FTICR-IMS imaging could be used to measure spatial heterogeneity in parasite lipid turnover within homogeneous monolayers of BMDMs. BMDMs were infected with L. mexicana and subsequently labeled with ^2^H_2_O for 96 h. Spatial heterogeneity over the scale of ∼100 µm was observed in ^2^H enrichment in parasite lipids (IPC d36:0 and PI *a*36:1) (Fig. 3C to E) and to a lesser extent in BMDM lipids such as PI 38:4 and ganglioside GM1b (Fig. 3E). These data indicate that the growth and/or metabolism of intracellular parasite stages may vary stochastically even under uniform culture conditions. This approach was extended to measure spatial heterogeneity in parasite lipid metabolism in acute phase granulomas following short-term ^2^H_2_O labeling to capture nonequilibrium labeling dynamics (Fig. 4A). While the overall level of ^2^H enrichment in parasite lipids showed a high level of consistency across whole granuloma sections, considerable heterogeneity was observed over short distances (Fig. 4B). In particular, ^2^H enrichment in parasite PI *a*36:1 and iM2 (adjusted to maximum enrichment of bulk lipids at 50 days) varied by 2- to 3-fold over distances of 100 to 400 µm (Fig. 4B). As expected, these differences were less apparent when labeling times were extended and ^2^H enrichment reached equilibrium, although some variability was still observed in lesions taken from mice that had been labeled for 15 or 50 days (Fig. 4B). Interestingly, labeling of parasite and host-derived PI molecular species was strongly correlated over a range of different time points from day 4 to day 12 (Fig. 4C). This correlation appeared to increase over time, indicating that short-term stochastic variation in parasite physiology is eventually dominated by host physiology and local changes in the tissue environment. Overall, these data support the conclusion that parasite growth and lipid metabolism in acute-phase granulomas exhibits considerable heterogeneity over shorter distances of a few tens to hundreds of µm. This heterogeneity may reflect stochastic differences in parasite growth and metabolism, as well as local differences in the tissue microenvironment and host cell physiology.

### Host cells harboring quiescent parasites accumulate in the underlying dermal layers.

L. mexicana induces large dermal granulomas in BALB/c mice that commonly extend into subcutaneous layers of the skin and the underlying mesothelium. The mesothelium constitutes an important basement membrane that separates the skin from the underlying muscle and adipose tissues. This layer is rich in collagen and stains strongly with Masson’s trichrome and periodic acid-Schiff stains (see Fig. S1). The mesothelium often expands around growing granulomas, reflecting active tissue repair and fibrosis (Fig. 4D). These fibrotic regions contained large numbers of infected host cells, based on diagnostic IMS mass signatures (Fig. 4E) and light microscopy of alternative sections (Fig. 4F). Strikingly, ^2^H enrichment in parasite lipids (IPC d36:0, PI *a*36:1, and iM2) in these regions was very low compared to central regions of the lesions (Fig. 4E and G, ROI 1 compared to ROIs 2, 3, and 4) and comparable to nondividing promastigotes (Fig. 3A). The expanded mesothelium layer may thus represent a specific niche that harbors nondividing or slow-dividing parasites in a metabolically quiescent state. To confirm that expansion of granulomatous lesions into underlying tissues is not just a feature of infections induced using high parasite infection doses and subcutaneous infection, we also investigated the histology of granulomas induced using a low parasite infection dose (100 parasites) and intradermal injection in the ear pinna. Ear granulomas were also observed to expand into and through the underlying collagen-rich cartilage layer (see Fig. S6), indicating that expansion of granulomas into nondermal tissues is not dependent on infection dose and injection route used.

10.1128/mBio.00129-21.6 FIG S6 Expansion of L. mexicana induced granulomas into underlying tissues in BALB/c ear pinna. Ear pinna of BALB/c mice were intradermally infected with 100 L. mexicana stationary-phase promastigotes. Swelling was detected after 4 weeks and mice culled and ear sections stained with Masson trichome. The large arrow indicates the cartilaginous layer along the medium section of the ear. Parasites were injected into the top side of the ear (granulomatous tissue outlined in dotted white line). Small arrows indicate prominent expansion of the granulomatous lesion into the bottom side of the ear across the collagen-rich layer of cartilage. Download FIG S6, TIF file, 1.6 MB. Copyright © 2021 Kloehn et al. 2021 Kloehn et al. https://creativecommons.org/licenses/by/4.0/ This content is distributed under the terms of the Creative Commons Attribution 4.0 International license.

### Miltefosine is concentrated in lesion tissues but not in developing fibrotic regions.

**FIG 5:**
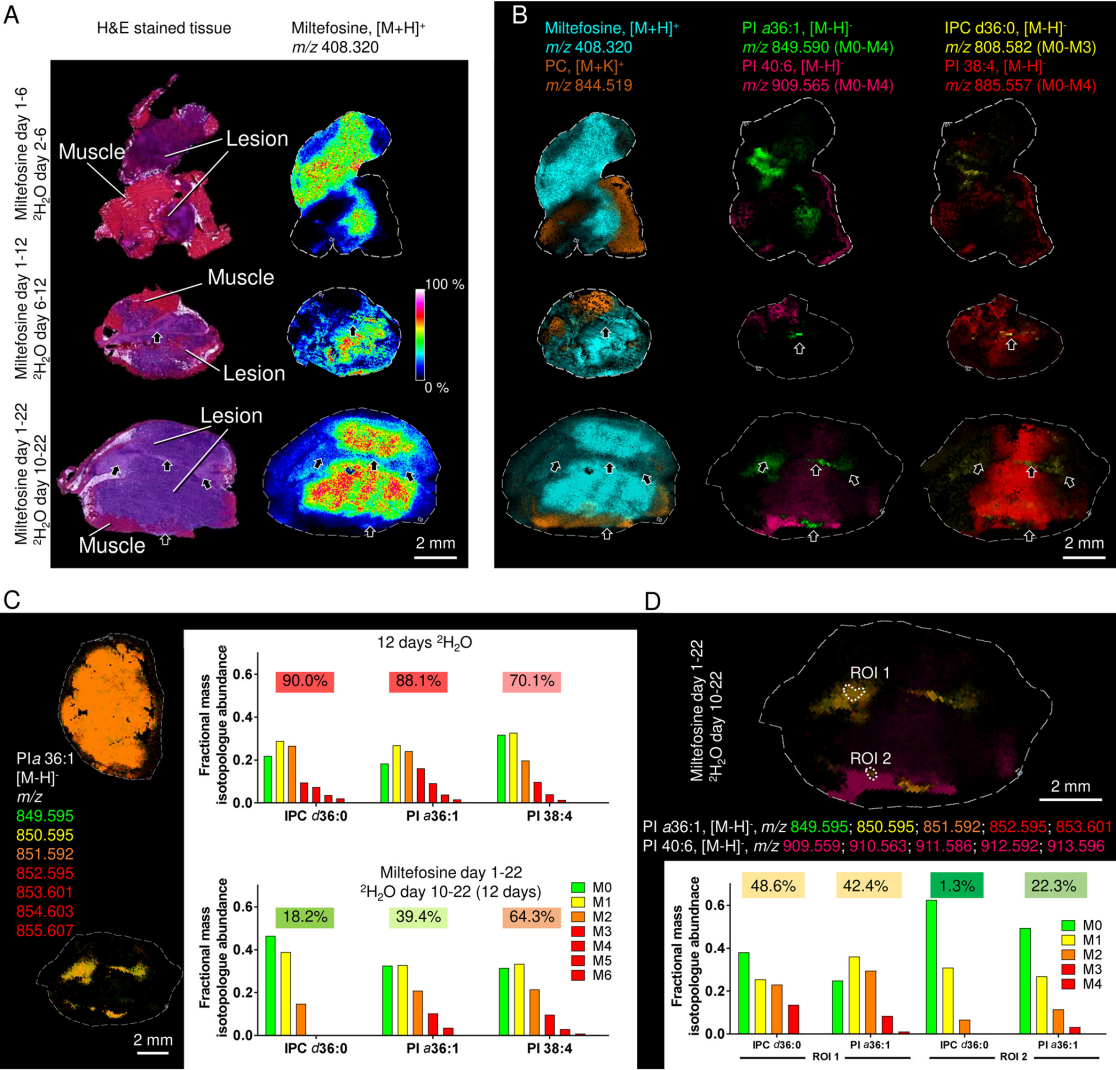
Association of quiescent parasite subpopulations with areas of fibrosis induced by miltefosine treatment. (A) L. mexicana-infected BALB/c mice were treated with miltefosine for 6, 12, or 22 days and granulomatous lesions sectioned for histology (H&E) and MALDI-FTICR-MS in positive- or negative-ion mode (50-μm spatial resolution in both cases). Miltefosine ([M+H]^+^ ion *m/z* 408.320) strongly accumulated in lesion tissue compared to surrounding muscle tissue and was excluded from the mesothelium and regions of lesion fibrosis that were evident by day 22. (B) Miltefosine and host PC species (*m/z* 844.519) detected in positive-ion MALDI-FTICR-MS define regions of lesion and nonlesion tissue, respectively. PI *a*36:1 and IPC d36:0 define populations of parasites that were initially cleared from miltefosine-rich regions but persisted in fibrotic tissues after 22 days of treatment. PI 40:6 and PI 38:4 represent host-specific lipids. (C) Mice were labeled at 5% ^2^H_2_O body water for 12 days without miltefosine treatment (upper panel) or with miltefosine treatment for 22 days (lower panel). The different mass isotopologues (M_0_ to M_6_) of the parasite-specific PI *a*36:0 were color-coded and merged to generate an isotopologue heat map. Average mass isotopologue distribution in parasite-specific lipids (IPC d36:0 and PI *a*36:1) and host lipid (PI 38:4) are shown, and the ^2^H labeling was quantified as the increase in average mass over natural abundance. (D) Lesion from drug-treated and ^2^H_2_O-labeled mouse as in panel C. The spatial distribution of muscle tissue-specific PI 40:6 (magenta) and parasite-specific PI *a*36:0 is shown. The different mass isotopologues (M_0_ to M_4_) are color coded and merged to generate an isotopologue heat map. The relative abundance of different mass isotopologues of the parasite-specific lipids, IPC d36:0 and PI *a*36:1, in two ROIs highlight heterogeneity within parasite populations during drug treatment.

Miltefosine is the only orally available drug used for the treatment of human visceral and cutaneous leishmaniasis. Treatment of cultured L. mexicana promastigotes with miltefosine leads to a dose-dependent inhibition of ^2^H_2_O incorporation into DNA and fatty acids (see Fig. S7A and B). Miltefosine has been shown to accumulate in the liver, lung, and kidney, although the extent to which it accumulates in granulomas has not been assessed (39). Miltefosine was detected in *Leishmania* granulomas as its protonated ion [M+H]^+^ using MALDI-FTICR-IMS and accumulated progressively over the course of the treatment (see Fig. S8A). Strikingly, accumulation was highest in granulomatous lesions compared to other organs/tissues, such as the spleen, liver, and lymph node (see Fig. S8B). Furthermore, the accumulation of miltefosine in the macrophage-rich core of acute phase granuloma contrasted with surrounding muscle and underlying mesothelial tissues, which had very low levels of miltefosine (Fig. 5A and B; see also Fig. S8B). Miltefosine was also readily detected in infected monolayers of BMDMs, indicating selective uptake or retention by macrophages (see Fig. S7C).

10.1128/mBio.00129-21.7 FIG S7 Dose-dependent effect of miltefosine on *Leishmania* viability and growth L. mexicana promastigotes (log phase) were treated with different concentrations of miltefosine (0 to 40 μM) in medium containing 5% ^2^H_2_O for 30 h. (A) DNA turnover was assessed by measuring rate of ^2^H incorporation into the deoxyribose moiety of DNA by GC-MS. (B) Fatty acid synthesis was assessed by measuring ^2^H enrichment in total cellular fatty acids, detected as their fatty acid methyl esters by GC-MS. *P* values were determined using a *t* test. Download FIG S7, TIF file, 0.5 MB. Copyright © 2021 Kloehn et al. 2021 Kloehn et al. https://creativecommons.org/licenses/by/4.0/ This content is distributed under the terms of the Creative Commons Attribution 4.0 International license.

10.1128/mBio.00129-21.8 FIG S8 Miltefosine accumulates efficiently in L. mexicana induced skin lesions. (A) *L. mexicana*-infected BALB/c mice were either given no treatment, a single dose of miltefosine (oral, 40 mg/kg) or daily treatment with miltefosine for 22 days (oral, 40 mg/kg/day). Lesions were excised and frozen and sections examined by H&E staining or analyzed by MALDI-FTICR-IMS in positive-ion mode at a 50-μm spatial resolution. All three tissues were analyzed in a single run, allowing direct comparison of the signal intensities. Miltefosine was not detected in the untreated lesion tissue, while low levels were detected in lesion tissue 24 h after a single dose. After 22 days of treatment, high levels of miltefosine are present in the lesion tissue. All intensities are displayed as a heat-map color gradient at 0 to 10% (% of maximum). This setting allows the visualization of miltefosine after a single dose but leads to saturation of the signal in the lesions from the 22 day treatment. (B) Several tissues (skin lesion, spleen, liver, and inguinal lymph node) were analyzed from a mouse after 12 days of miltefosine treatment (oral, 40 mg/kg/day). Sections were H&E stained, and subsequent sections were analyzed by MALDI-FTICR-IMS at 50-μm spatial resolution (50 × 50-μm laser spot/data point) in positive mode. All tissues were analyzed in a single run allowing direct comparison of the signal intensity. All intensities are displayed as a heatmap color gradient at 0 to 30% (% of maximum). The highest levels of miltefosine were detected in granulomatous tissues (black arrow, white outline). No miltefosine was detected in the adjacent muscle tissue (white arrows, black outline). The lower levels of miltefosine in the lymph node could indicate another safe reservoir for the parasites. (C) L. mexicana-infected BMM monolayers were treated with 0 or 10 μM miltefosine for 36 h, and freeze-dried monolayers were coated with matrix and analyzed by MALDI-FTICR-IMS. Miltefosine [M+H]^+^ was readily detected in treated but not in untreated BMMs. As a reference, the distribution and intensity of an unidentified lipid is shown (*m/z* 851.651 in positive mode). Download FIG S8, TIF file, 2.6 MB. Copyright © 2021 Kloehn et al. 2021 Kloehn et al. https://creativecommons.org/licenses/by/4.0/ This content is distributed under the terms of the Creative Commons Attribution 4.0 International license.

Miltefosine treatment led to a reduction in the lesion size and appearance of collagen-rich regions indicative of tissue repair (arrows in Fig. 5A; see also Fig. S1B and C). H&E and Masson trichrome staining, as well as MALDI-FTICR-IMS (Fig. 5B), showed that the collagen-rich regions in resolving lesions retained parasites, while the original lesion tissue was largely cleared of infected cells. Strikingly, and in contrast to the granulomatous tissues, the collagen-rich regions did not accumulate miltefosine (Fig. 5A; see also Fig. S1C). Parasites in these tissues also appeared to be in a slow-growth/metabolically quiescent state, since the *in vivo* labeling with ^2^H_2_O (over 6 to 12 days) was markedly reduced compared to average labeling of parasite lipids in non-drug-treated animals (Fig. 5C; see also Fig. S9**)**. In contrast, the turnover of host lipids (e.g., PI 38:4) in the same tissues were essentially unchanged following miltefosine treatment (Fig. 5C). Together, these data demonstrate that miltefosine preferentially accumulates in macrophage-rich regions of acute-phase lesions harboring metabolically active parasites. In contrast, miltefosine fails to accumulate in the underlying mesothelium layer or newly deposited collagen-rich tissues in resolving lesions which harbor metabolically quiescent parasites. Spatial differences in miltefosine accumulation, coupled with differences in the physiological state of resident parasites, may thus account for the persistence of some parasite populations during drug treatment.

10.1128/mBio.00129-21.9 FIG S9 Heterogeneous parasite populations during miltefosine treatment. L. mexicana infected mice were treated with miltefosine for 6 days (A) or 12 days (B) before being labeled with ^2^H_2_O (4 and 6 days, respectively). The spatial distribution of muscle-specific PI 40:6 (magenta) and parasite-specific PI *a*36:0 is indicated. The different mass isotopologues (M_0_ to M_4_) are color-coded and merged to generate an isotopologue heat map. The relative abundance of different mass isotopologues of the parasite-specific lipids IPC d36:0 and PI *a*36:1 in two ROIs highlight heterogeneity within parasite populations during drug treatment. Download FIG S9, TIF file, 2.3 MB. Copyright © 2021 Kloehn et al. 2021 Kloehn et al. https://creativecommons.org/licenses/by/4.0/ This content is distributed under the terms of the Creative Commons Attribution 4.0 International license.

## DISCUSSION

There is increasing evidence that heterogeneity in both microbial growth rate and metabolism allows pathogens to adapt to local changes in different tissue microenvironments, as well as to resist drug treatments (1, 40). However, measurement of microbial metabolic/growth heterogeneity *in vivo*, particularly for slow-growing pathogens, is challenging. Recent studies have provided evidence for heterogeneity in *Leishmania* growth in granulomatous tissues based on the use of parasites expressing photoactivatable fluorescent proteins or *in vivo* BrdU labeling of DNA (20, 25, 30, 41). While these approaches allow measurement of protein or DNA turnover at the single cell level, they cannot be used to determine absolute rates of protein turnover/cell replication and require generation of transgenic parasite lines or labeling of parasites *in vivo* with dyes that may not equilibrate equally across all tissues. They also provide no information on the physiological state of host cells in the same tissue microenvironment. In this study, we have coupled *in vivo* ^2^H_2_O stable isotope labeling with imaging mass spectrometry (IMS) to measure the temporal-spatial dynamics of parasite and host lipid dynamics in *Leishmania* granulomas. ^2^H_2_O rapidly equilibrates across all tissues, with concomitant incorporation of deuterium into a wide range of primary and secondary metabolites, as well as major cellular macromolecules (31, 42). We show that this approach can be used to determine spatial differences in the absolute rates of turnover of complex plasma membrane lipids, providing a proxy for both the rate of growth and metabolic state of both the pathogen and the host cells in infected tissues. We show that the L. mexicana granulomas contain a mosaic of metabolically active and semiquiescent parasites during acute phases of infection. Unexpectedly, we identified a distinct population of infected host cells harboring quiescent parasites in the collagen-rich mesothelium that separates the skin from underlying tissues. Expansion of these collagen-rich tissues during miltefosine treatment appears to provide a safe haven for viable, but metabolically quiescent parasites, which are exposed to lower concentrations of miltefosine than in surrounding tissues. These data suggest that temporal-spatial changes in the granuloma tissue environment during drug treatments may lead to the expansion of metabolically quiescent parasite populations in tissue niches with sublethal drug levels, providing an explanation for treatment failure.

MALDI-FTICR-IMS allowed the detection of approximately 5,000 and >10,000 molecular features in negative- and positive-ion modes, respectively (data not shown). Here, we have focused on lipid species detected using negative ion FTICR-MS, since these included both parasite-specific inositol lipids (PI, IPC, and GIPLs), as well as host-specific gangliosides, which are strongly ionized and readily detected under these conditions. The rates of ^2^H enrichment in these lipids in cultured promastigote stages appeared to reflect both the rate of cell division and the metabolic state of different stages and the basal rate of organelle and plasma membrane turnover. Similarly, the levels of ^2^H enrichment in tissue amastigote lipids are also likely to reflect both the growth rate and metabolic state of these stages. In particular, the average *t*_1/2_ of lesion amastigote lipids was approximately twice as fast as the rate of DNA replication (∼6 versus 12 days) (31), whereas MS/MS analysis of parasite iM2 indicated that deuterium was incorporated via multiple pathways in hexose and lipid metabolism. ^2^H enrichment in macrophage host lipids is also likely to reflect the replicative capacity, as well as differences in the metabolic or activation state of these cells. However, it is important to note that monocytes are continuously recruited to *Leishmania* granulomas and actively phagocytose preapoptotic infected host cells (41), resulting in the internalization of a large bolus of labeled lipids (43). As such, the rate of labeling of host lipids likely represents the maximum rate of replication and/or maintenance turnover.

Our results show that parasite growth and metabolism within granulomas varies considerably across distances of 10 to a few 100 µm. This could reflect stochastic variability and/or differences in the tissue microenvironment. Stochastic events have been shown to trigger some bacteria to switch between active growth and nongrowing, persister states under otherwise uniform growth conditions (1). Spontaneous reversible switching between normal and persister cells may constitute a bet-hedging strategy that improves bacterial survival under variable growth conditions (44). However, the extent to which eukaryotic pathogens, such as *Leishmania*, utilize the same strategy is less well defined (40, 45). We found evidence for stochastic changes in parasites growth/metabolism from analysis of ^2^H enrichment in amastigote lipids in cultured BMDMs. Alternatively, local gradients in nutrients, oxygen, host microbicidal factors, and cytokines on the scale of a few 100 µm could also contribute to heterogeneity in macrophage growth, polarization, and permissiveness for parasite growth. A number of factors are thought to contribute to local heterogeneity in *Leishmania* granulomas. For example, the production of diffusible NO by recently recruited macrophage/monocytes may suppress parasite growth in proximal cells, as well as the inhibition of host cell oxidative metabolism and the production of a wide range of inflammatory cytokines and chemokines (26). On the other hand, excessive NO production eventually leads to inhibition of the local inflammatory responses and reduced NO production, creating local feedback loops. In support of local gradients of NO generating regions of both parasite and host cell inhibition, we observed a strong correlation between ^2^H incorporation into parasite and host lipids across granuloma profiles (Fig. 4C). Although decreased lipid synthesis may reflect a general inhibition of parasite/host cell growth, it is also worth noting that NO is a potent inhibitor of the tricarboxylic acid cycle, which provides essential precursors, such as citrate, for lipid synthesis. As such, analysis of ^2^H incorporation into lipids may provide a sensitive readout of NO levels. Alternatively, heterogeneity within *Leishmania* granulomas could also reflect the local production of anti-inflammatory cytokines (i.e., IL-4) by granuloma eosinophils, which polarizes infected macrophages toward a parasite permissive M2 phenotype, even in the presence of a strong host inflammatory response (21). Intriguingly, the M2 polarized monocytes/macrophages produce chemokines that promote eosinophil recruitment providing a second feedback mechanism for sustaining heterogeneous host responses and parasite growth within granuloma tissues (21). Therefore, temporal-spatial changes in host cell dynamics, together with other nutrient/oxygen gradients, may create distinct niches on a scale of ∼100 µm within granulomatous tissues that contribute to the heterogeneity in parasite lipid turnover.

We showed that acute-phase granulomatous lesions can span multiple dermal layers in susceptible BALB/c mice and penetrate to the underlying mesothelium, a collagen-rich basement layer that demarks the transition from skin to underlying muscle tissues (46). This is not an exclusive feature of high-infection-dose subcutaneous infections, since similar expansion of granulomatous tissue into underlying layers was also observed in low-infection-dose intradermal infections of the ear pinna. The mesothelium harbored a distinct population of parasites that are metabolically quiescent, exhibiting negligible rates of ^2^H incorporation into cellular lipids. Turnover of host lipids was slightly elevated in these regions, which may reflect the involvement of these cells in the deposition of collagen-rich extracellular matrix, a process that is generally associated with the upregulation of arginase-1 and other enzymes involved in polyamine and proline biosynthesis (47). Although the upregulation of host arginase-1 can promote intracellular growth of *Leishmania* amastigotes by increasing availability of polyamines, the finding that *Leishmania* parasites in this zone are metabolically quiescent suggests that other factors, such as limiting nutrient levels, anoxia, or elevated levels of reactive oxygen species, may restrict parasite growth. Interestingly, a recent analysis of L. major growth rates in latently infected murine tissues also found a population of nondividing parasites in arginase-1-positive host cells (30), supporting the conclusion that multiple factors control intracellular parasite growth.

We also used ^2^H_2_O-MALDI-FTICR-IMS to investigate the tissue distribution and parasite response to the front-line anti-leishmanial drug, miltefosine. Miltefosine is the only orally available drug for human leishmaniasis and is proposed to target metabolic processes in both host macrophages (48) and intracellular parasite stages (6, 49). Miltefosine was shown to be highly enriched in lesion granulomas compared to surrounding tissues (dermal, muscle, and adipose), which likely contributes to the efficacy of this drug. Miltefosine treatment decreased the parasite burden, leading to a reduction in lesion size with concomitant deposition of collagen. Intriguingly, the collagen-rich tissues in resolving lesions failed to accumulate miltefosine and contained host cells that were infected with quiescent parasites. These studies suggest that the metabolically quiescent parasites in the mesothelium layer of acute lesions or the collagen-rich tissues of drug-treated lesions may be less susceptible to miltefosine and/or that their persistence following miltefosine treatment reflects a lack of drug accumulation in these tissue niches. These findings are consistent with human studies showing that miltefosine treatment often results in nonsterile cure and high risk of disease reactivation (13–15, 50, 51). Combination treatments designed to increase the effectiveness of miltefosine should therefore include a partner drug that target quiescent parasite stages and/or are able to access collagen-rich tissue regions in resolving lesions.

In summary, we show that measurement of spatial-temporal changes in lipid dynamics in *Leishmania* granulomas using ^2^H_2_O labeling and MALDI-FTICR-IMS can be used to detect changes in the tissue microenvironment and host-pathogen interactions *in vivo*. Specifically, we have identified significant heterogeneity in parasite lipid turnover in acutely infected tissues, likely reflecting stochastic or tissue microenvironment effects on parasite growth and metabolism. We also identified a distinct host niche in the collagen-rich mesothelium basement membranes harboring metabolically quiescent parasites that may persist during natural healing and drug treatment. More broadly, this approach is applicable to analyzing the metabolic state and drug responses of other microbial pathogens *in vivo* (23, 40, 52–54).

## MATERIALS AND METHODS

### Promastigote culture, ^2^H_2_O labeling, and harvest.

L. mexicana promastigotes (<20 passages in culture since isolation from infected tissues) were cultured in 10 ml of RPMI 1640 medium (Gibco Life Sciences) (pH 7.4) supplemented with 10% (vol/vol) heat-inactivated fetal calf serum (FCS). For labeling analyzes, ^2^H_2_O was spiked as phosphate-buffered saline made up in 100% ^2^H_2_O (^2^H_2_O-PBS) into the cell culture medium. For MALDI-FTICR-IMS analysis, cells were harvested (500 × *g*, 10 min, 4°C) and washed twice with ice-cold PBS, and the pellets were stored at –80°C.

### Macrophage infection and ^2^H_2_O labeling.

J774 macrophages were cultured in 250-ml/75-cm^2^ flasks (Corning Life Sciences) in RPMI 1640 medium (Gibco Life Sciences) (pH 7.4) supplemented with 10% (vol/vol) heat-inactivated FCS. Confluent monolayers were detached using a cell scraper and the cell pellets obtained by centrifugation (500 × *g*, 10 min, 4°C), washed twice in ice-cold PBS, and stored at −80°C prior to extraction. BMDMs were derived from the femurs of BALB/c mice and cultured in RPMI supplemented with 20% (vol/vol) L-cell medium, 15% FCS (vol/vol), and penicillin-streptomycin (100 U/ml; Life Science Technologies). BMDMs (2 × 10^5^) were seeded in 1 ml of medium into each well of a Millicell EZ slide (4-well glass). The following day, the cells were infected with L. mexicana stationary-phase promastigotes (50 μl, 1.5 × 10^6^ cells) for 24 h before being washed three times with PBS to remove extracellular parasites and resuspended in fresh media. At 3 days postinfection, the cell culture medium was replaced by medium containing 5% H_2_O-PBS or ^2^H_2_O-PBS, with or without miltefosine (10 μM). Infected cells were labeled/treated for 3 days and then washed five times with PBS and once with 40 mM ammonium acetate. The residual liquid was carefully wicked off the Millicell EZ slide, and the slides were frozen on a piece of foil floating on liquid nitrogen. The frozen slides were freeze-dried overnight and stored in a vacuum desiccator until further processing.

### Mouse infections, ^2^H_2_O labeling, drug treatment, and tissue harvest.

The use of mice for this study was approved by The University of Melbourne Animal Ethics Committee. Female BALB/c mice were maintained at 21 to 23°C on a 14-h/10-h light/dark cycle with free access to standard rodent chow diet and drinking water. Mice (6 to 7 weeks of age) were routinely infected subcutaneously in the rump with 10^6^ L. mexicana stationary-phase promastigotes in 50 µl of PBS. Cutaneous lesions (5 to 10 mm in diameter) typically formed 60 to 90 days after initiation of infection. In the experiment described in Fig. S6, the ear pinnae of BALB/c mice were intradermally infected with 100 L. mexicana promastigotes in 10 µl of PBS (low-dose, intradermal infection).

Mice were metabolically labeled with ^2^H_2_O during the acute phase of infection when rump lesions were ∼5 mm in diameter. Labeling was initiated by intraperitoneal injection with 99.9% ^2^H_2_O made up to 0.9% NaCl (35 μl of ^2^H_2_O/g of body weight), and then mice were provided with 9% ^2^H_2_O in their drinking water for the duration of the experiment (33). This regime results in a stable enrichment of the body water at 5% ^2^H_2_O (31, 33, 55). Acutely infected mice (5-mm lesions) were treated with miltefosine, which was administered orally by gavage (40 mg/kg in 200 μl of H_2_O) for short treatments (single dose or 6 days of treatment) or given in the drinking water for longer treatments (40 mg/kg/day, assuming an average water intake of 5 ml per day per mouse [56]). Body weight and overall well-being were monitored daily throughout the treatment to ensure that water intake was not affected by the addition of miltefosine. Mice were culled humanely and the granulomatous lesion carefully excised, including surrounding skin and muscle tissue. Skin lesions and other organs were frozen on aluminum foil floating on liquid nitrogen and stored at −80°C until further processing.

### IMS sample preparation.

Frozen cell pellets (L. mexicana promastigotes, J774 macrophages) and tissue samples (granulomas, liver, spleen, and lymph node) were mounted onto a microtome chuck using optimal cutting temperature compound (O.C.T.; Tissue-Tek). Samples were sectioned on a Reichardt-Jung Frigocut 2800N cryotome modified to use tungsten-steel microtome blades (Feather C35). Sections were cut at 12-µm thickness, gently transferred onto prechilled glass slides (Menzel-Glaeser Superfrost Ultra Plus Glass slides) using a fine painting brush, and freeze-thaw mounted by gently warming from the rear of the slide. Mounted sections were vacuum dried in a desiccator until further processing. The MALDI matrix compound, 1,8-bis-(1-pyrrolidinyl)naphthalene (BPYN), was applied as described previously (57) with minor modifications as outlined here. A saturated solution BPYN (ca. 3 to 5 mg/ml, made up in 100% acetonitrile) was pipetted onto sections of cell pellets (1 µl per application) and allowed to dry for 5 min at room temperature before reapplication (6× to 8×). Matrix coated cell pellets were dried in a desiccator for at least 1 h prior to analysis by MALDI-FTICR-IMS. Tissues were coated with a fine, even layer of BPYN matrix using a TM-Sprayer matrix application device (HTX Technologies LLC) with an attached LC20-AD HPLC pump (Shimadzu Scientific Instruments) for wet matrix deposition using the following settings: a flow rate of 0.15 ml/min, a gas flow rate of 10 liters/min, and a nozzle temperature of 84°C. Spray conditions were as follows: eight passes, a nozzle velocity of 900 mm/min, and track spacing of 2 mm, with alternate passes set at 90°C offset and repeat passes set to an offset of 1 mm. Prepared sections were vacuum dried in a desiccator for at least 1 h prior to analysis, and the even application of matrix crystals was assessed by light microscopy.

### MALDI-FTICR-IMS analysis.

MALDI-FTICR-IMS was performed using a Bruker SolariX 7T Hybrid electrospray ionization/matrix assisted laser desorption ionization–Fourier transform ion cyclotron resonance mass spectrometer. The instrument was operated in negative ion mode using optimized instrumental settings for the mass range *m/z* 200/300 to 2,000 in broadband mode with a 2 megaword time domain providing an estimated resolving power of 130,000 at *m/z* 400. For MALDI-FTICR-IMS, the instrument was operated in the negative-ion mode with the laser set to 30% power for BPYN matrix using a minimum spot size of 25 to 30 µm, smart-walk enabled with a random pattern, in either a 50 × 50-µm or 75 × 75-µm spot array, using between 250 and 500 laser shots. Mass spectra from MALDI-FTICR-IMS analyses were viewed using Compass Data Analysis, and images were viewed and generated using flexImaging (Bruker). The intensity of all viewed masses was set to 10 to 70% unless indicated otherwise. Lipids were identified based on their accurate mass and database searches (lipidmaps.org) (58). Searches were performed for the ion [M-H]^−^ at a mass tolerance of 0.01 (*m/z*) or higher. MS/MS analyses were conducted on select lipid species directly off tissue/slide using the following settings: between 5 and 10,000 laser shots and a collision energy of 60 or 70 V.

### Histology.

For H&E staining, frozen lesions were sectioned and dried as described above. Alternatively, rump or ear pinna lesions were chemically fixed in 5 ml of formalin solution (neutral buffered, 10%; Sigma-Aldrich) overnight and embedded in paraffin, and 5-μm sections were prepared. Fixed tissues were stained with H&E or Masson trichome (collagen) using protocols established by the University of Melbourne Histology Facility.

### Flow cytometry.

Cutaneous lesions were excised as before, together with equivalent sized cutaneous samples, distal to the lesion, which served as an internal control (nonlesion). Single-cell suspensions were prepared by disruption of samples with collagenase and DNase using standard protocols, followed by filtration through 70-µm strainers to remove debris. Cells were stained with fixable/viability dye eFl506 (Thermo Fisher) for identification of viable cells and then labeled with a panel of antibodies conjugated to a range of fluorophores: anti-CD3 BV421 (17A2), anti-Ly6C BV605 (AL-21), anti-MHC II BV786 (M5/114), anti-CD4 FITC (GK1.5), anti-Siglec F PerCP-Cy5.5 (E50-2440), anti-F4/80 PE (T45-2342), anti-CD11c PE-Cy7 (HL3), anti-CD11b A647 (M1/70), anti-Ly6G A700 (1A8), anti-CD45.2 eFl780 (104). Data were acquired on a LSR Fortessa (BD Biosciences) and analyzed using FlowJo Software (TreeStar, Inc., Ashland, OR). The data are presented following exclusion of doublets.

### GC-MS analysis of fatty acids and DNA derived deoxyribose.

Various concentrations of miltefosine (0, 2.5, 5, 10, 20, and 40 μM) were added to log-phase L. mexicana promastigote cultures.^2^H_2_O-PBS (final concentration, 5% [vol/vol]) was added 1 h after initiation of drug treatment, and the cells were incubated for 30 h. Cells were quenched and lysed, and metabolites were extracted using a chloroform-methanol-water extraction, as described previously (59). Fatty acids and DNA derived deoxyribose were prepared and analyzed by gas chromatography-mass spectrometry (GC-MS), and ^2^H labeling was quantified as the excess molar enrichment of the M1 mass isotopologue over the parental M0 ion for each fatty acid, as described previously (31, 33).

## References

[1] Bumann D. 2015. Heterogeneous host-pathogen encounters: act locally, think globally. Cell Host Microbe 17:13–19. doi:10.1016/j.chom.2014.12.006. 25590757

[2] Claudi B, Sprote P, Chirkova A, Personnic N, Zankl J, Schurmann N, Schmidt A, Bumann D. 2014. Phenotypic variation of *Salmonella* in host tissues delays eradication by antimicrobial chemotherapy. Cell 158:722–733. doi:10.1016/j.cell.2014.06.045. 25126781

[3] Cohen NR, Lobritz MA, Collins JJ. 2013. Microbial persistence and the road to drug resistance. Cell Host Microbe 13:632–642. doi:10.1016/j.chom.2013.05.009. 23768488 PMC3695397

[4] Helaine S, Cheverton AM, Watson KG, Faure LM, Matthews SA, Holden DW. 2014. Internalization of Salmonella by macrophages induces formation of nonreplicating persisters. Science 343:204–208. doi:10.1126/science.1244705. 24408438 PMC6485627

[5] Manina G, Dhar N, McKinney JD. 2015. Stress and host immunity amplify *Mycobacterium tuberculosis* phenotypic heterogeneity and induce nongrowing metabolically active forms. Cell Host Microbe 17:32–46. doi:10.1016/j.chom.2014.11.016. 25543231

[6] Burza S, Croft SL, Boelaert M. 2018. Leishmaniasis. Lancet 392:951–970. doi:10.1016/S0140-6736(18)31204-2. 30126638

[7] Alvar J, Velez ID, Bern C, Herrero M, Desjeux P, Cano J, Jannin J, den Boer M, Team WHOLC. 2012. Leishmaniasis worldwide and global estimates of its incidence. PLoS One 7:e35671. doi:10.1371/journal.pone.0035671. 22693548 PMC3365071

[8] Pisarski K. 2019. The global burden of disease of zoonotic parasitic diseases: top 5 contenders for priority consideration. Trop Med Infect Dis 4:44. doi:10.3390/tropicalmed4010044. PMC6473363 30832380

[9] Banuls AL, Bastien P, Pomares C, Arevalo J, Fisa R, Hide M. 2011. Clinical pleiomorphism in human leishmaniases, with special mention of asymptomatic infection. Clin Microbiol Infect 17:1451–1461. doi:10.1111/j.1469-0691.2011.03640.x. 21933304

[10] Kaye PM, Cruz I, Picado A, Van Bocxlaer K, Croft SL. 2020. Leishmaniasis immunopathology-impact on design and use of vaccines, diagnostics and drugs. Semin Immunopathol 42:247–264. doi:10.1007/s00281-020-00788-y. 32152715

[11] Calvopina M, Gomez EA, Sindermann H, Cooper PJ, Hashiguchi Y. 2006. Relapse of new world diffuse cutaneous leishmaniasis caused by *Leishmania* (*Leishmania*) *mexicana* after miltefosine treatment. Am J Trop Med Hyg 75:1074–1077. doi:10.4269/ajtmh.2006.75.1074. 17172368

[12] Zerpa O, Ulrich M, Blanco B, Polegre M, Avila A, Matos N, Mendoza I, Pratlong F, Ravel C, Convit J. 2007. Diffuse cutaneous leishmaniasis responds to miltefosine but then relapses. Br J Dermatol 156:1328–1335. doi:10.1111/j.1365-2133.2007.07872.x. 17441955

[13] Vanaerschot M, Dumetz F, Roy S, Ponte-Sucre A, Arevalo J, Dujardin JC. 2014. Treatment failure in leishmaniasis: drug resistance or another (epi-) phenotype? Expert Rev Anti Infect Ther 12:937–946. doi:10.1586/14787210.2014.916614. 24802998

[14] Dorlo TP, Rijal S, Ostyn B, de Vries PJ, Singh R, Bhattarai N, Uranw S, Dujardin JC, Boelaert M, Beijnen JH, Huitema AD. 2014. Failure of miltefosine in visceral leishmaniasis is associated with low drug exposure. J Infect Dis 210:146–153. doi:10.1093/infdis/jiu039. 24443541

[15] Deep DK, Singh R, Bhandari V, Verma A, Sharma V, Wajid S, Sundar S, Ramesh V, Dujardin JC, Salotra P. 2017. Increased miltefosine tolerance in clinical isolates of *Leishmania donovani* is associated with reduced drug accumulation, increased infectivity and resistance to oxidative stress. PLoS Negl Trop Dis 11:e0005641. doi:10.1371/journal.pntd.0005641. 28575060 PMC5470736

[16] Murray HW. 2001. Tissue granuloma structure-function in experimental visceral leishmaniasis. Int J Exp Pathol 82:249–267. doi:10.1046/j.1365-2613.2001.00199.x. 11703536 PMC2517779

[17] Salguero FJ, Garcia-Jimenez WL, Lima I, Seifert K. 2018. Histopathological and immunohistochemical characterization of hepatic granulomas in *Leishmania donovani*-infected BALB/c mice: a time-course study. Parasit Vectors 11:73. doi:10.1186/s13071-018-2624-z. 29386047 PMC5793367

[18] Saunders EC, McConville MJ. 2020. Immunometabolism of *Leishmania* granulomas. Immunol Cell Biol 98:832–844. doi:10.1111/imcb.12394. 32780446

[19] Kaye PM, Beattie L. 2016. Lessons from other diseases: granulomatous inflammation in leishmaniasis. Semin Immunopathol 38:249–260. doi:10.1007/s00281-015-0548-7. 26678994 PMC4779128

[20] Carneiro MB, Lopes ME, Hohman LS, Romano A, David BA, Kratofil R, Kubes P, Workentine ML, Campos AC, Vieira LQ, Peters NC. 2020. Th1-Th2 cross-regulation controls early *Leishmania* infection in the skin by modulating the size of the permissive monocytic host cell reservoir. Cell Host Microbe 27:752–768 e7. doi:10.1016/j.chom.2020.03.011. 32298657

[21] Lee SH, Chaves MM, Kamenyeva O, Gazzinelli-Guimaraes PH, Kang B, Pessenda G, Passelli K, Tacchini-Cottier F, Kabat J, Jacobsen EA, Nutman TB, Sacks DL. 2020. M2-like, dermal macrophages are maintained via IL-4/CCL24-mediated cooperative interaction with eosinophils in cutaneous leishmaniasis. Sci Immunol 5:eaaz4415. 32276966 10.1126/sciimmunol.aaz4415 PMC7385908

[22] Hurrell BP, Beaumann M, Heyde S, Regli IB, Muller AJ, Tacchini-Cottier F. 2017. Frontline science: *Leishmania mexicana* amastigotes can replicate within neutrophils. J Leukoc Biol 102:1187–1198. doi:10.1189/jlb.4HI0417-158R. 28798144

[23] Ramakrishnan L. 2012. Revisiting the role of the granuloma in tuberculosis. Nat Rev Immunol 12:352–366. doi:10.1038/nri3211. 22517424

[24] Sacks D, Noben-Trauth N. 2002. The immunology of susceptibility and resistance to *Leishmania major* in mice. Nat Rev Immunol 2:845–858. doi:10.1038/nri933. 12415308

[25] Muller AJ, Aeschlimann S, Olekhnovitch R, Dacher M, Spath GF, Bousso P. 2013. Photoconvertible pathogen labeling reveals nitric oxide control of *Leishmania major* infection *in vivo* via dampening of parasite metabolism. Cell Host Microbe 14:460–467. doi:10.1016/j.chom.2013.09.008. 24139402

[26] Postat J, Olekhnovitch R, Lemaitre F, Bousso P. 2018. A metabolism-based quorum-sensing mechanism contributes to termination of inflammatory responses. Immunity 49:654–665. doi:10.1016/j.immuni.2018.07.014. 30266340

[27] Schatz V, Neubert P, Rieger F, Jantsch J. 2018. Hypoxia, hypoxia-inducible factor-1α, and innate antileishmanial immune responses. Front Immunol 9:216. doi:10.3389/fimmu.2018.00216. 29520262 PMC5827161

[28] Jantsch J, Schatz V, Friedrich D, Schroder A, Kopp C, Siegert I, Maronna A, Wendelborn D, Linz P, Binger KJ, Gebhardt M, Heinig M, Neubert P, Fischer F, Teufel S, David JP, Neufert C, Cavallaro A, Rakova N, Kuper C, Beck FX, Neuhofer W, Muller DN, Schuler G, Uder M, Bogdan C, Luft FC, Titze J. 2015. Cutaneous Na^+^ storage strengthens the antimicrobial barrier function of the skin and boosts macrophage-driven host defense. Cell Metab 21:493–501. doi:10.1016/j.cmet.2015.02.003. 25738463 PMC4350016

[29] Romano A, Carneiro MBH, Doria NA, Roma EH, Ribeiro-Gomes FL, Inbar E, Lee SH, Mendez J, Paun A, Sacks DL, Peters NC. 2017. Divergent roles for Ly6C^+^ CCR2^+^ CX3CR1^+^ inflammatory monocytes during primary or secondary infection of the skin with the intra-phagosomal pathogen *Leishmania major*. PLoS Pathog 13:e1006479. doi:10.1371/journal.ppat.1006479. 28666021 PMC5509374

[30] Mandell MA, Beverley SM. 2017. Continual renewal and replication of persistent *Leishmania major* parasites in concomitantly immune hosts. Proc Natl Acad Sci U S A 114:E801–E810. doi:10.1073/pnas.1619265114. 28096392 PMC5293024

[31] Kloehn J, Saunders EC, O’Callaghan S, Dagley MJ, McConville MJ. 2015. Characterization of metabolically quiescent Leishmania parasites in murine lesions using heavy water labeling. PLoS Pathog 11:e1004683. doi:10.1371/journal.ppat.1004683. 25714830 PMC4340956

[32] Sernee MF, Ralton JE, Nero TL, Sobala LF, Kloehn J, Vieira-Lara MA, Cobbold SA, Stanton L, Pires DEV, Hanssen E, Males A, Ward T, Bastidas LM, van der Peet PL, Parker MW, Ascher DB, Williams SJ, Davies GJ, McConville MJ. 2019. A family of dual-activity glycosyltransferase-phosphorylases mediates mannogen turnover and virulence in *Leishmania* parasites. Cell Host Microbe 26:385–399. doi:10.1016/j.chom.2019.08.009. 31513773

[33] Kloehn J, McConville MJ. 2020. Analysis of the physiological and metabolic state of leishmania using heavy water labeling. Methods Mol Biol 2116:587–609. doi:10.1007/978-1-0716-0294-2_35. 32221944

[34] Hsu FF, Turk J, Zhang K, Beverley SM. 2007. Characterization of inositol phosphorylceramides from *Leishmania major* by tandem mass spectrometry with electrospray ionization. J Am Soc Mass Spectrom 18:1591–1604. doi:10.1016/j.jasms.2007.05.017. 17627842 PMC2065762

[35] De Castro Levatti EV, Toledo MS, Watanabe Costa R, Bahia D, Mortara RA, Takahashi HK, Straus AH. 2017. *Leishmania* (*Viannia*) *braziliensis* inositol phosphorylceramide: distinctive sphingoid base composition. Front Microbiol 8:1453. doi:10.3389/fmicb.2017.01453. 28824583 PMC5543781

[36] Ralton JE, McConville MJ. 1998. Delineation of three pathways of glycosylphosphatidylinositol biosynthesis in *Leishmania mexicana*: precursors from different pathways are assembled on distinct pools of phosphatidylinositol and undergo fatty acid remodeling. J Biol Chem 273:4245–4257. doi:10.1074/jbc.273.7.4245. 9461623

[37] McConville MJ, Blackwell JM. 1991. Developmental changes in the glycosylated phosphatidylinositols of *Leishmania donovani*: characterization of the promastigote and amastigote glycolipids. J Biol Chem 266:15170–15179. doi:10.1016/S0021-9258(18)98600-X. 1831200

[38] Yohe HC, Macala LJ, Giordano G, McMurray WJ. 1992. GM1b and GM1b-GalNAc: major gangliosides of murine-derived macrophage-like WEHI-3 cells. Biochim Biophys Acta 1109:210–217. doi:10.1016/0005-2736(92)90085-z. 1520698

[39] Breiser A, Kim DJ, Fleer EA, Damenz W, Drube A, Berger M, Nagel GA, Eibl H, Unger C. 1987. Distribution and metabolism of hexadecylphosphocholine in mice. Lipids 22:925–926. doi:10.1007/BF02535556.]. 3444386

[40] Barrett MP, Kyle DE, Sibley LD, Radke JB, Tarleton RL. 2019. Protozoan persister-like cells and drug treatment failure. Nat Rev Microbiol 17:607–620. doi:10.1038/s41579-019-0238-x. 31444481 PMC7024564

[41] Heyde S, Philipsen L, Formaglio P, Fu Y, Baars I, Höbbel G, Kleinholz CL, Seiss EA, Stettin J, Gintschel P, Dudeck A, Bousso P, Schraven B, Müller AJ. 2018. CD11c-expressing Ly6C^+^ CCR2^+^ monocytes constitute a reservoir for efficient *Leishmania* proliferation and cell-to-cell transmission. PLoS Pathog 14:e1007374. doi:10.1371/journal.ppat.1007374. 30346994 PMC6211768

[42] Louie KB, Bowen BP, McAlhany S, Huang Y, Price JC, Mao JH, Hellerstein M, Northen TR. 2013. Mass spectrometry imaging for in situ kinetic histochemistry. Sci Rep 3:1656. doi:10.1038/srep01656. 23584513 PMC3625901

[43] Zhang S, Weinberg S, DeBerge M, Gainullina A, Schipma M, Kinchen JM, Ben-Sahra I, Gius DR, Yvan-Charvet L, Chandel NS, Schumacker PT, Thorp EB. 2019. Efferocytosis fuels requirements of fatty acid oxidation and the electron transport chain to polarize macrophages for tissue repair. Cell Metab 29:443–456. doi:10.1016/j.cmet.2018.12.004. 30595481 PMC6471613

[44] Pontes MH, Groisman EA. 2020. A physiological basis for nonheritable antibiotic resistance. mBio 11:e00817-20. doi:10.1128/mBio.00817-20. 32546621 PMC7298711

[45] Dumoulin PC, Burleigh BA. 2018. Stress-induced proliferation and cell cycle plasticity of intracellular *Trypanosoma cruzi* amastigotes. mBio 9:e00673-18. doi:10.1128/mBio.00673-18. 29991586 PMC6050952

[46] Melo F, Amaral M, Oliveira P, Lima W, Andrade M, Michalick M, Raso P, Tafuri W, Tafuri W. 2008. Diffuse intralobular liver fibrosis in dogs naturally infected with *Leishmania* (*Leishmania*) *chagasi*. Am J Trop Med Hyg 79:198–204. doi:10.4269/ajtmh.2008.79.198. 18689624

[47] Muxel SM, Aoki JI, Fernandes JCR, Laranjeira-Silva MF, Zampieri RA, Acuna SM, Muller KE, Vanderlinde RH, Floeter-Winter LM. 2017. Arginine and polyamines fate in leishmania infection. Front Microbiol 8:2682. doi:10.3389/fmicb.2017.02682. 29379478 PMC5775291

[48] Zhang N, Prasad S, Huyghues Despointes CE, Young J, Kima PE. 2018. *Leishmania* parasitophorous vacuole membranes display phosphoinositides that create conditions for continuous Akt activation and a target for miltefosine in *Leishmania* infections. Cell Microbiol 20:e12889. doi:10.1111/cmi.12889. 29993167 PMC6202129

[49] Bhattacharya A, Bigot S, Padmanabhan PK, Mukherjee A, Coelho A, Leprohon P, Papadopoulou B, Ouellette M. 2020. New insights in the mode of action of antileishmanial drugs by using chemical mutagenesis screens coupled to next-generation sequencing. Microb Cell 7:59–61. doi:10.15698/mic2020.02.708. 32025514 PMC6993126

[50] Coelho AC, Trinconi CT, Costa CH, Uliana SR. 2016. *In vitro* and *in vivo* miltefosine susceptibility of a *Leishmania amazonensis* isolate from a patient with diffuse cutaneous leishmaniasis: follow-up. PLoS Negl Trop Dis 10:e0004720. doi:10.1371/journal.pntd.0004720. 27416021 PMC4945063

[51] Rai K, Cuypers B, Bhattarai NR, Uranw S, Berg M, Ostyn B, Dujardin JC, Rijal S, Vanaerschot M. 2013. Relapse after treatment with miltefosine for visceral leishmaniasis is associated with increased infectivity of the infecting *Leishmania donovani* strain. mBio 4:e00611-13. doi:10.1128/mBio.00611-13. PMC3791894 24105765

[52] Blanc L, Lenaerts A, Dartois V, Prideaux B. 2018. Visualization of mycobacterial biomarkers and tuberculosis drugs in infected tissue by MALDI-MS imaging. Anal Chem 90:6275–6282. doi:10.1021/acs.analchem.8b00985. 29668262 PMC5956283

[53] Cicchese JM, Dartois V, Kirschner DE, Linderman JJ. 2020. Both pharmacokinetic variability and granuloma heterogeneity impact the ability of the first-line antibiotics to sterilize tuberculosis granulomas. Front Pharmacol 11:333. doi:10.3389/fphar.2020.00333. 32265707 PMC7105635

[54] Trindade S, Rijo-Ferreira F, Carvalho T, Pinto-Neves D, Guegan F, Aresta-Branco F, Bento F, Young SA, Pinto A, Van Den Abbeele J, Ribeiro RM, Dias S, Smith TK, Figueiredo LM. 2016. *Trypanosoma brucei* parasites occupy and functionally adapt to the adipose tissue in mice. Cell Host Microbe 19:837–848. doi:10.1016/j.chom.2016.05.002. 27237364 PMC4906371

[55] Busch R, Neese RA, Awada M, Hayes GM, Hellerstein MK. 2007. Measurement of cell proliferation by heavy water labeling. Nat Protoc 2:3045–3057. doi:10.1038/nprot.2007.420. 18079703

[56] Bachmanov AA, Reed DR, Beauchamp GK, Tordoff MG. 2002. Food intake, water intake, and drinking spout side preference of 28 mouse strains. Behav Genet 32:435–443. doi:10.1023/a:1020884312053. 12467341 PMC1397713

[57] Boughton BA, Thomas ORB, Demarais NJ, Trede D, Swearer SE, Grey AC. 2020. Detection of small molecule concentration gradients in ocular tissues and humours. J Mass Spectrom 55:e4460. doi:10.1002/jms.4460. 31654531

[58] Fahy E, Cotter D, Byrnes R, Sud M, Maer A, Li J, Nadeau D, Zhau Y, Subramaniam S. 2007. Bioinformatics for lipidomics. Methods Enzymol 432:247–273. doi:10.1016/S0076-6879(07)32011-9. 17954221

[59] Saunders EC, Ng WW, Kloehn J, Chambers JM, Ng M, McConville MJ. 2014. Induction of a stringent metabolic response in intracellular stages of *Leishmania mexicana* leads to increased dependence on mitochondrial metabolism. PLoS Pathog 10:e1003888. doi:10.1371/journal.ppat.1003888. 24465208 PMC3900632

