# Supplementary figures and images for: Identification of Metabolically Quiescent Leishmania mexicana Parasites in Peripheral and Cured Dermal Granulomas Using Stable Isotope Tracing Imaging Mass Spectrometry
Source: mBio. 2021 Apr 6;12(2):e00129-21. doi: 10.1128/mBio.00129-21 (PMC8092208; doi:10.1128/mBio.00129-21)

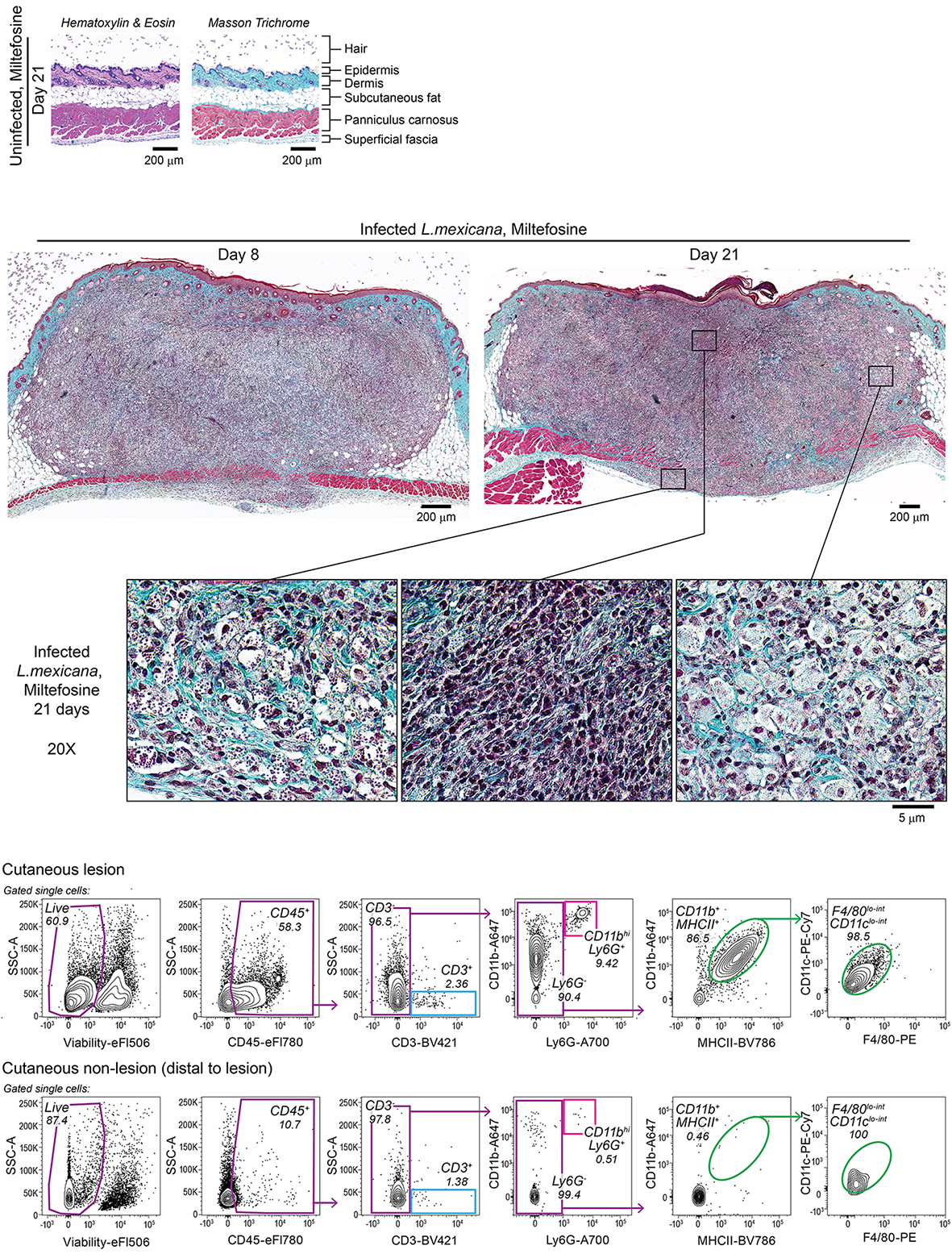

Supplement: FIG S1 [file mBio.00129-21-sf001.tif]

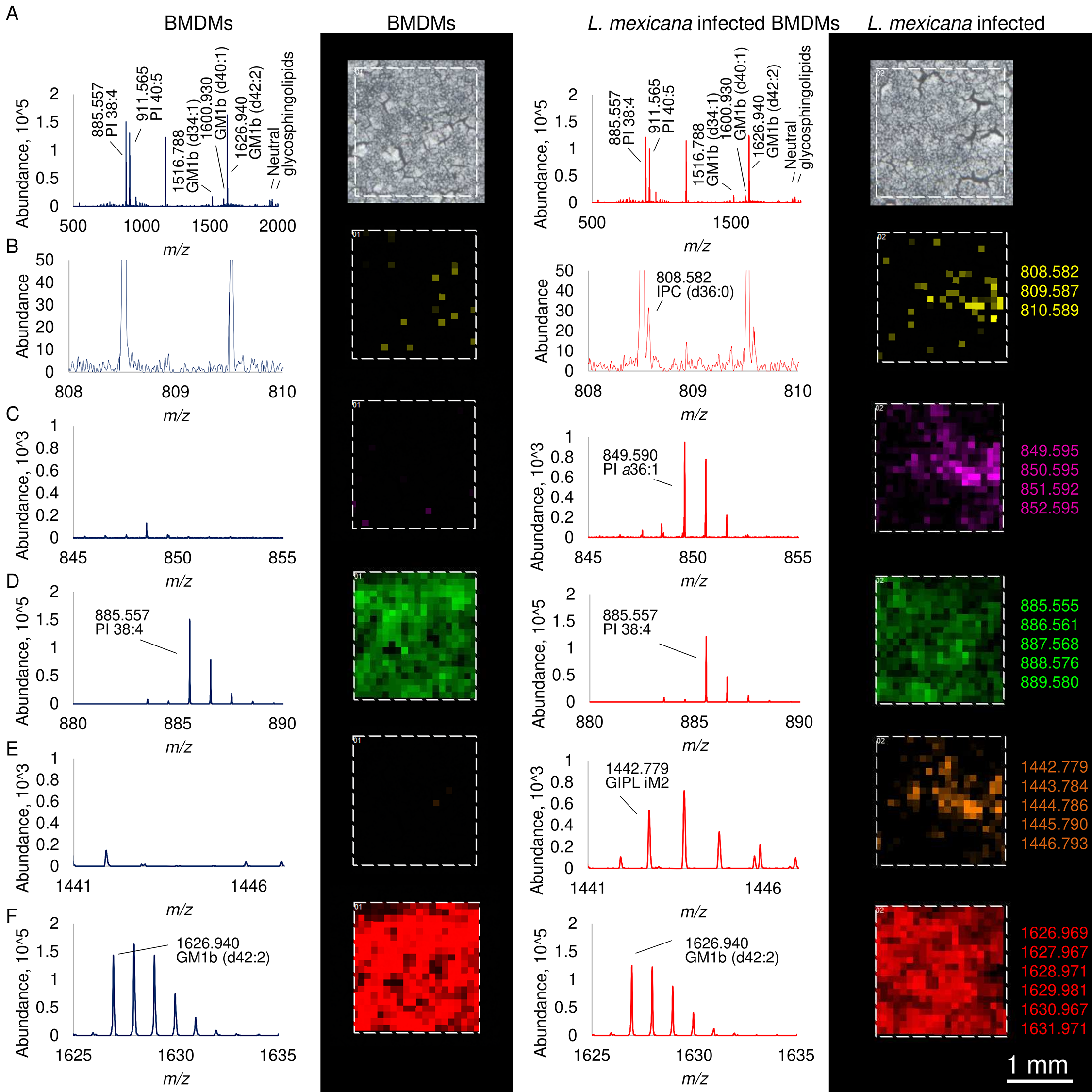

Supplement: FIG S2 [file mBio.00129-21-sf002.tif]

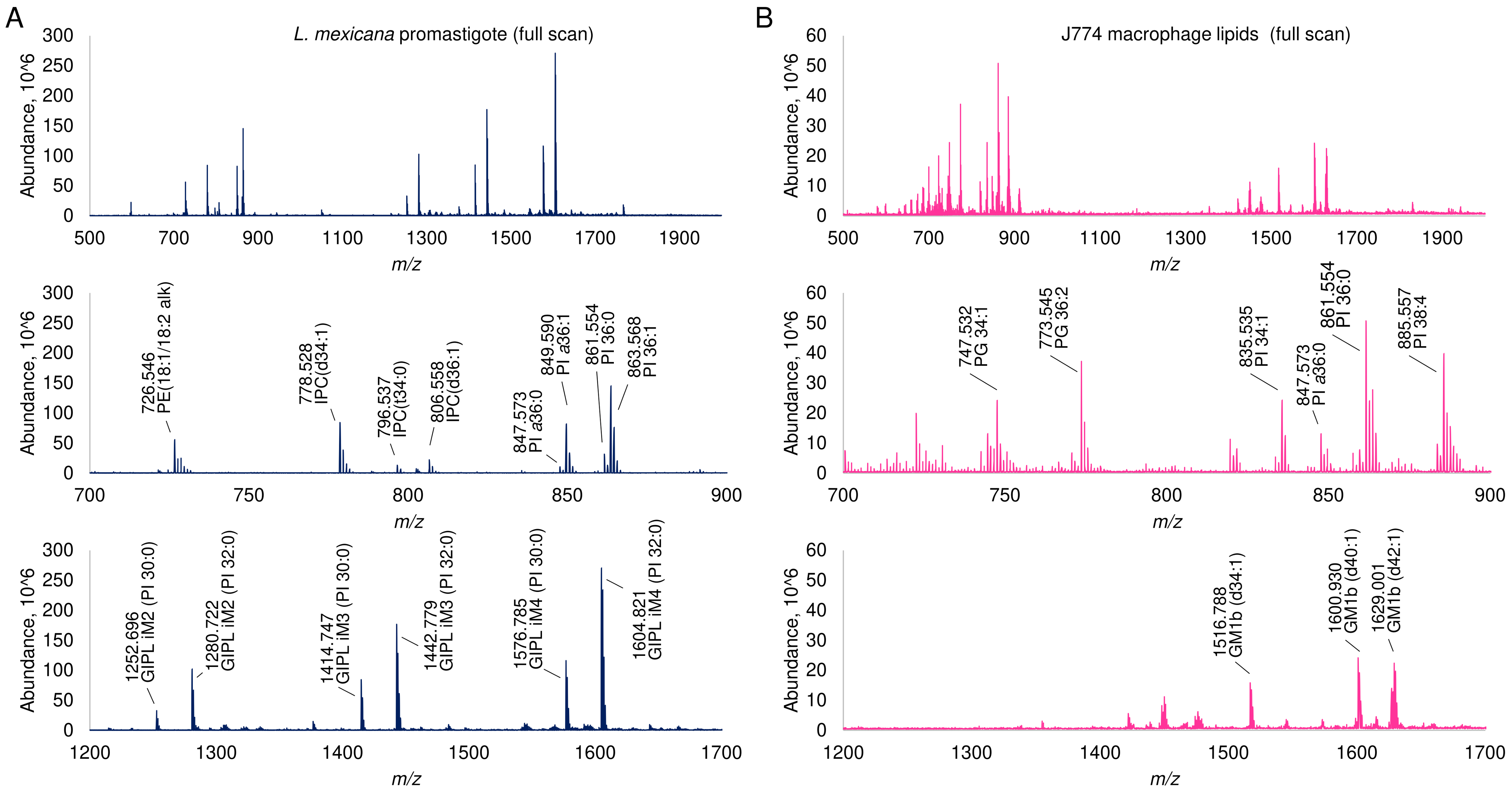

Supplement: FIG S3 [file mBio.00129-21-sf003.tif]

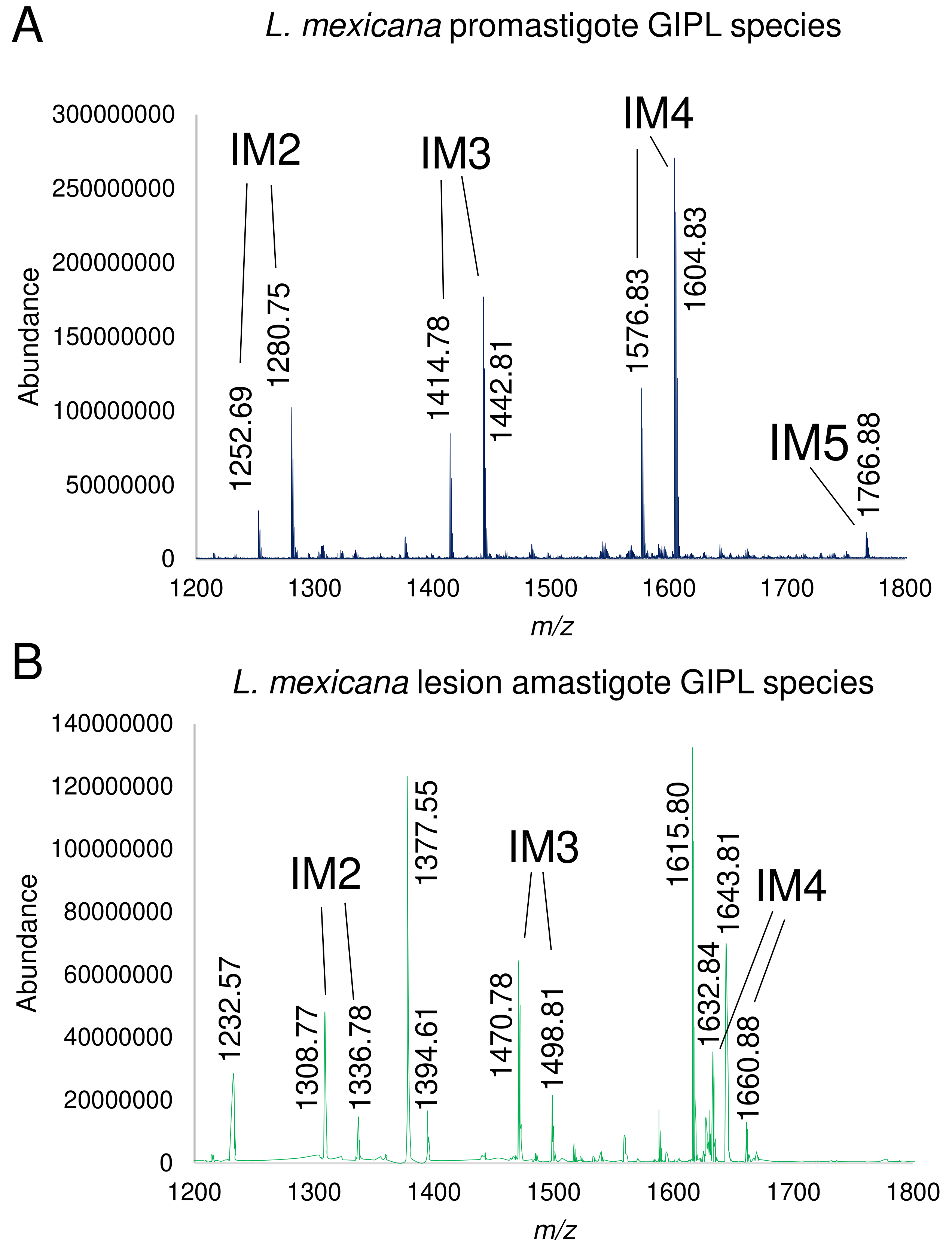

Supplement: FIG S4 [file mBio.00129-21-sf004.tif]

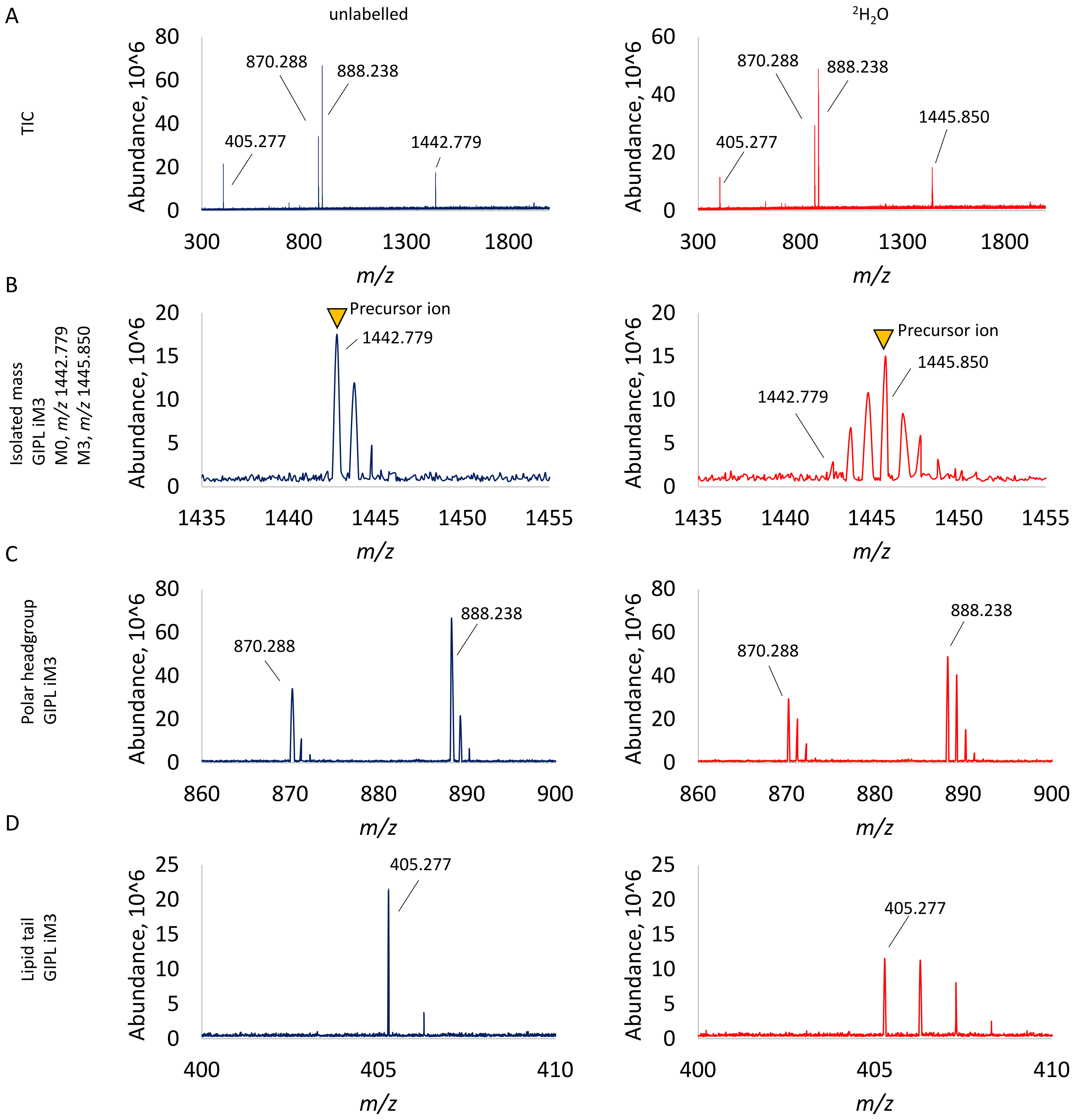

Supplement: FIG S5 [file mBio.00129-21-sf005.tif]

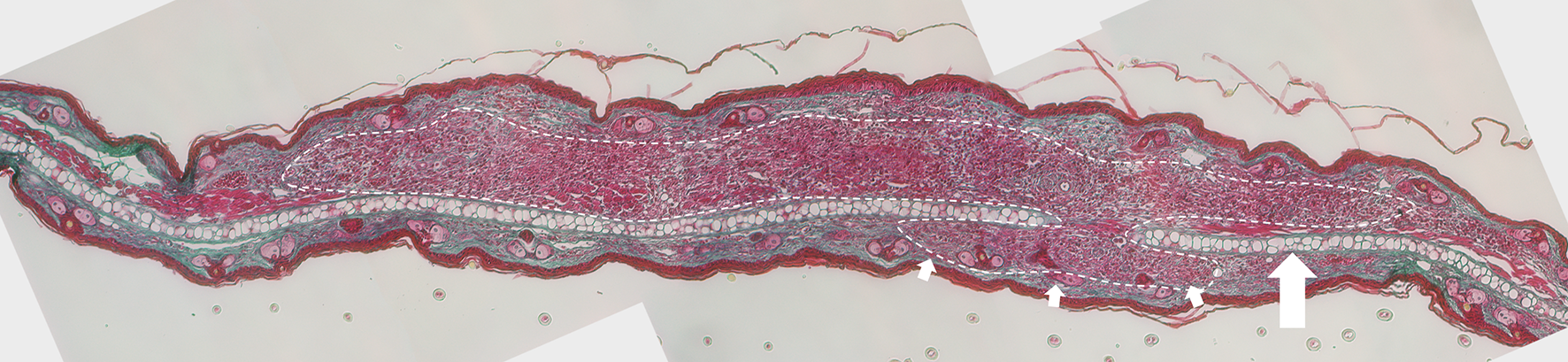

Supplement: FIG S6 [file mBio.00129-21-sf006.tif]

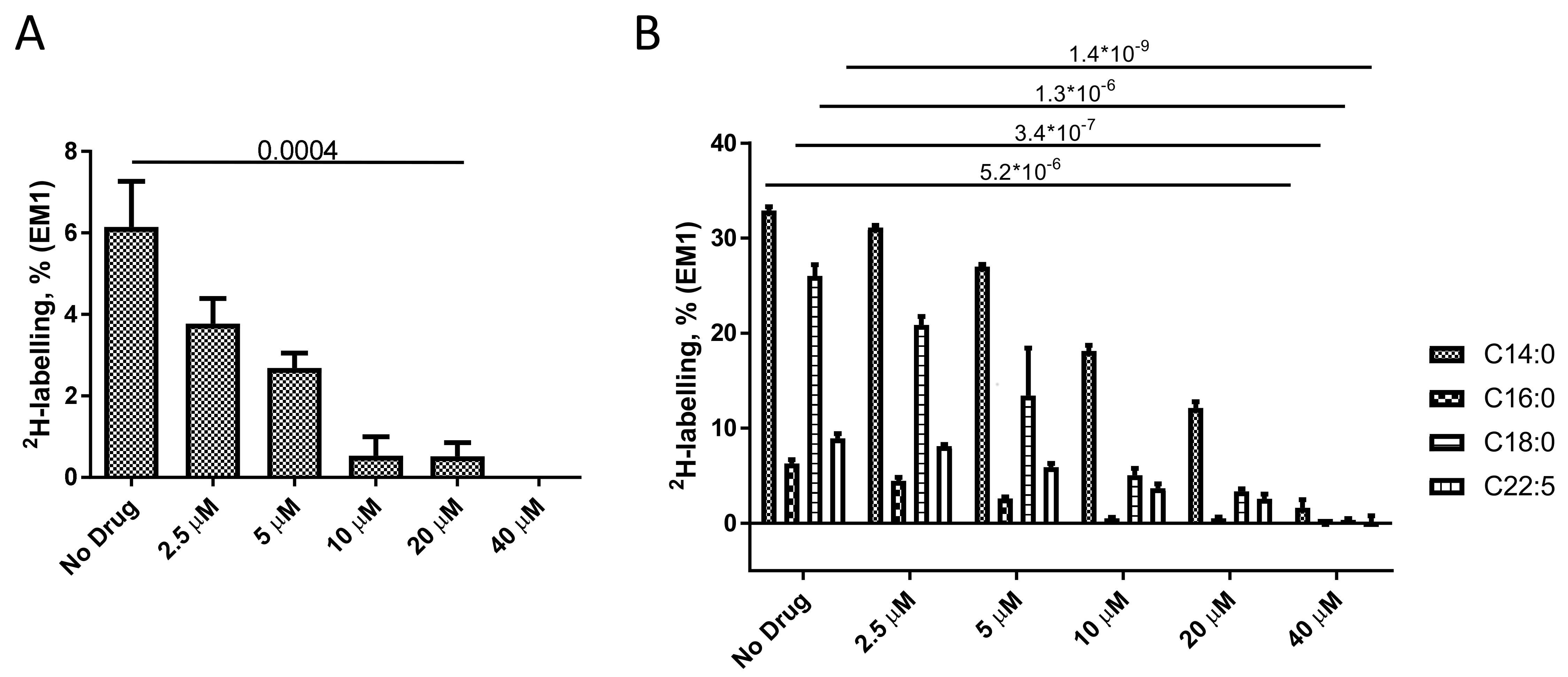

Supplement: FIG S7 [file mBio.00129-21-sf007.tif]

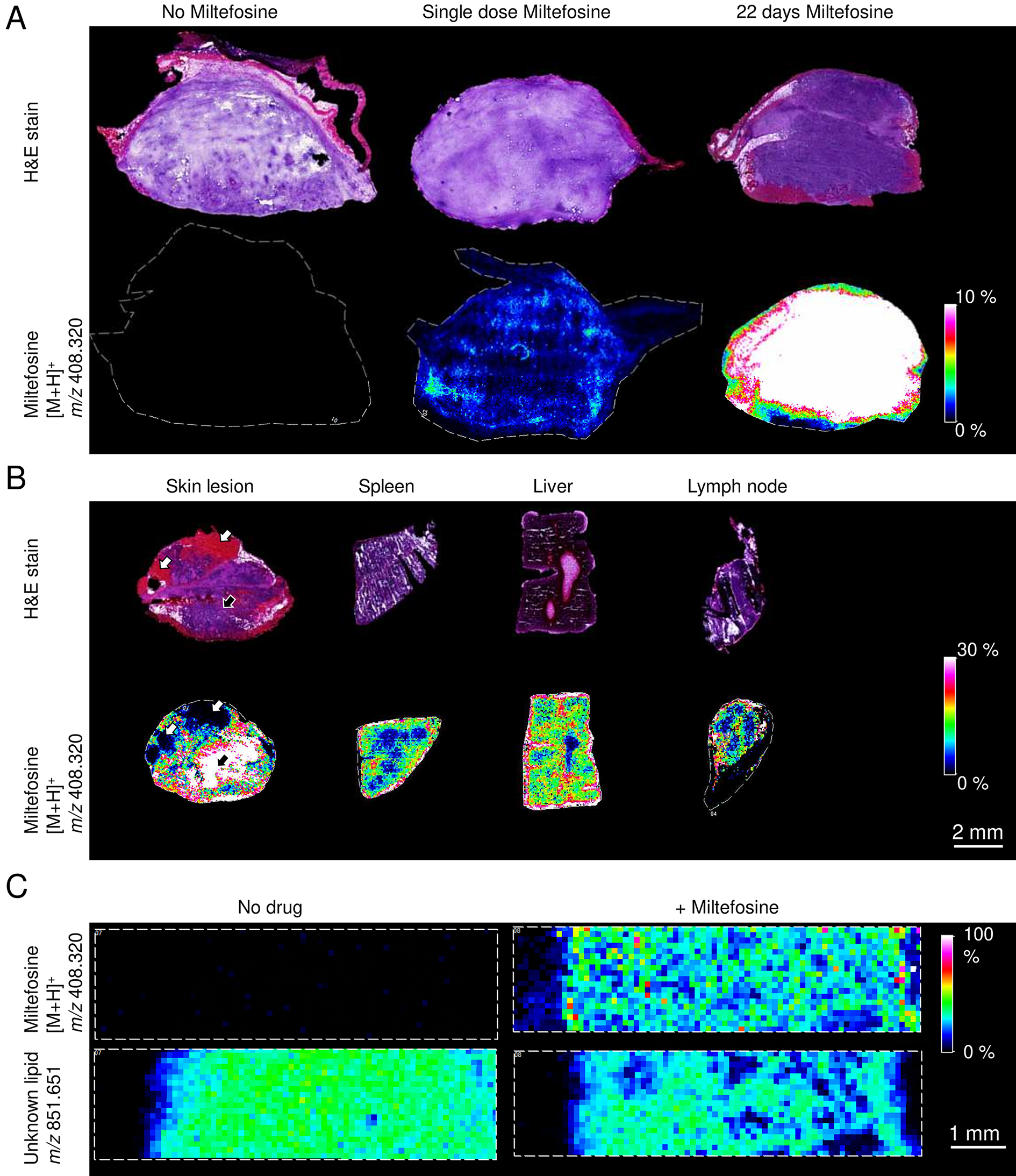

Supplement: FIG S8 [file mBio.00129-21-sf008.tif]

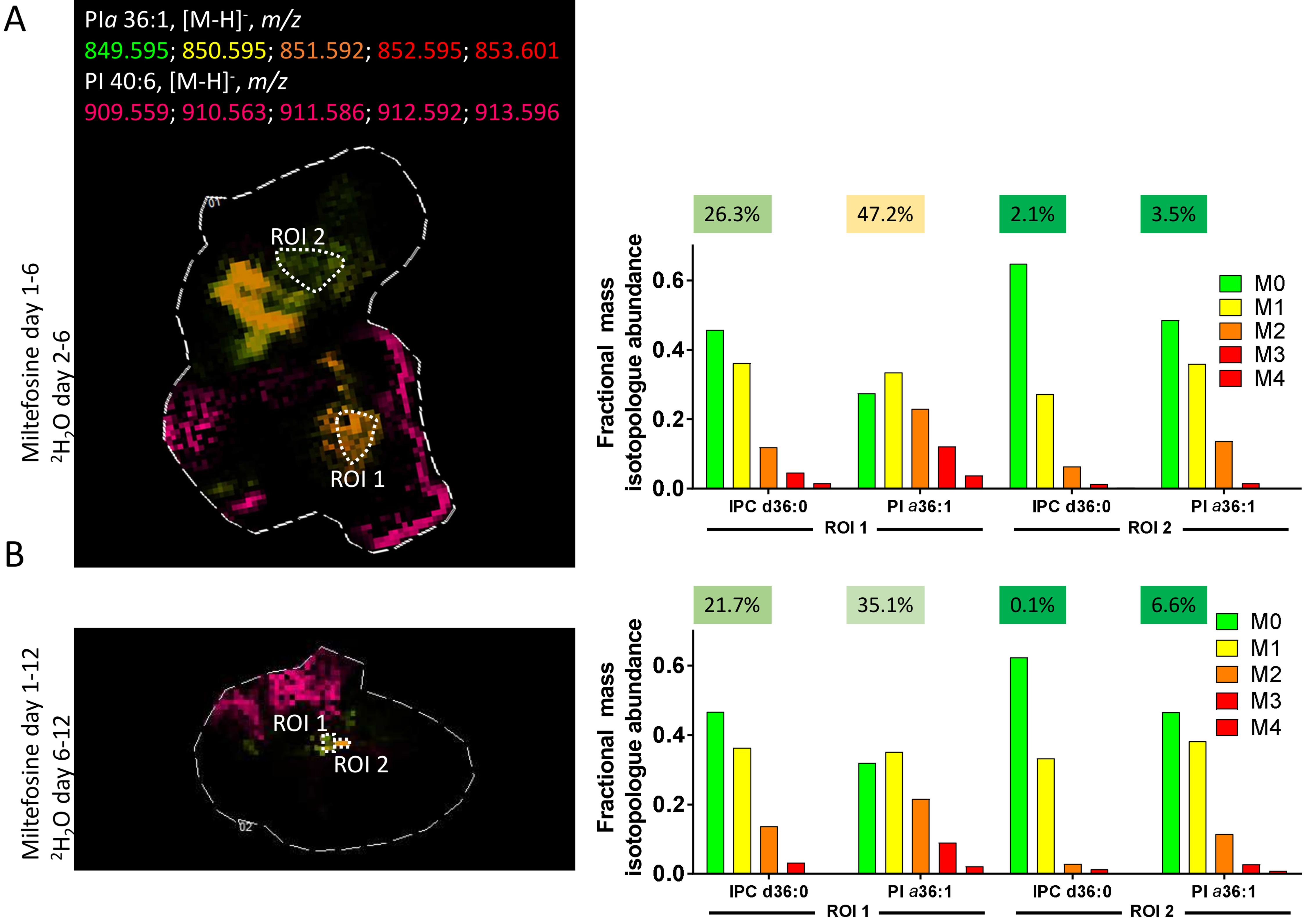

Supplement: FIG S9 [file mBio.00129-21-sf009.tif]
